# Taming Interfacial Ion‐Dipole Interactions With *d*‐Orbital Delocalized Electron Catalysis Expediates Low‐Temperature Li Metal Batteries

**DOI:** 10.1002/adma.202510894

**Published:** 2025-10-11

**Authors:** Jing Zhang, Fangqi Liu, Rong He, Qinghua Guan, Na Tian, Jian Wu, Zhenjiang Cao, Shikai Yin, Yongzheng Zhang, Lujie Jia, Xifei Li, Caiyin You, Haitao Liu, Meinan Liu, Yidong Miao, Hongzhen Lin, Jian Wang

**Affiliations:** ^1^ School of Materials Science and Engineering Xi'an University of Technology Xi'an 710048 China; ^2^ *i*‐Lab and CAS Key Laboratory of Nanophotonic Materials and Devices Suzhou Institute of Nano‐tech and Nano‐bionics Chinese Academy of Sciences Suzhou 215123 China; ^3^ Helmholtz Institute Ulm (HIU) D89081 Ulm Germany; ^4^ Karlsruhe Institute of Technology (KIT) D76021 Karlsruhe Germany; ^5^ College of Advanced Interdisciplinary Studies National University of Defense Technology Changsha 410073 China; ^6^ School of Textile & Clothing Nantong University Nantong 226019 China; ^7^ School of Chemistry Engineering Research Center of Energy Storage Materials and Devices Xi'an Jiaotong University Xi'an Shaanxi 710049 China; ^8^ Institute of Applied Physics and Computational Mathematics Beijing 100088 China; ^9^ School of Materials and Chemical Engineering Xuzhou University of Technology Xuzhou 221018 China

**Keywords:** delocalization‐electron modulation, interfacial desolvation kinetics, ion‐dipole interaction, lithium metal battery, low‐temperature battery

## Abstract

Low‐temperature lithium metal batteries (LT‐LMBs) are increasingly desired for higher energy density and longer lifespan. However, due to organic electrolyte solidification, LT‐LMBs are impeded by huge barriers resulting from the hindrance of larger solvation shells with strong ion‐dipole interactions, leading to depressive Li kinetics and severe dendrite formation. Herein, interfacial catalysis by constructing electron delocalization *d*‐orbital metal oxides toward the ion‐dipole interactions is pioneered to accelerate the larger Li(solvents)*_x_* ^+^ dissociation under the low‐temperature environment. Specifically, various kinds of *d*‐orbital metal oxides (M = Ti, V, Fe, Co) with oxygen defect modulation are systematically screened and investigated for breaking the ion‐dipole interactions, and the prototyped titanium oxide with adjustable electron delocalization behave the best, as confirmed by electrochemical and theoretical experiments. Consequently, optimized Li electrodes withstand environmental robustness from 25 to −50°C, and stabilize long‐term cycling up to 1800 h and high Coulombic efficiency without any short‐circuit under −20°C. The as‐fabricated Li–S full cell enables a high‐capacity retention of 88% at 0.2 C over 200 cycles, and the high‐loading Li‐LiNi_0.8_Co_0.1_Mn_0.1_O_2_ cell (≈20 mg cm^−2^) demonstrates excellent capacity retention ≈100% under 0°C, providing a new guideline for adopting a catalytic strategy for achieving advanced LT‐LMBs.

## Graphical abstract

The ion‐dipole interactions within Li(solvents)*_x_* ^+^ groups for fast interfacial Li^+^ desolvation/diffusion and dendrite‐free deposition are reduced by taming various *d*‐orbital electron‐delocalized catalyzers under the low‐temperature surroundings. Consequently, the cells with weak ion‐dipole interaction delivers long‐term cycling life with high environmental robustness from 25 to −50°C, and the practical full cell stabilizes excellent capacity retention of ≈100% under 0°C.

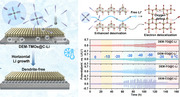

## 1 Introduction

In comparison to commercial graphite anode, lithium metal electrode is an ideal anode for achieving high‐energy‐density Li batteries because of the ultrahigh capacity (3860 mA h g^−1^) and the lowest electrochemical potential (−3.04 V vs standard hydrogen electrode).^[^ ^1^, ^2^, ^3^ ^]^ The electrochemical performance of Li metal batteries is of significance under extreme temperatures, especially under low temperature around −50°C.^[^ ^4^, ^5^ ^]^ However, the low‐temperature Li metal batteries (LT‐LMBs) suffer from uncontrollable Li dendrite growth, the uneven formation of solid‐electrolyte interphase (SEI) and electrolyte solidification, which are detrimental to pose safety hazards.^[^ ^6^, ^7^, ^8^, ^9^ ^]^ As known, the Li ion is the main charge carrier and the plating behavior contains several steps from interfacial desolvation of breaking Li^+^‐solvent interaction to electrochemical reduction to Li atoms, and final to subsequent migration of these atoms to potential nucleation sites.^[^ ^10^, ^11^, ^12^ ^]^ The failure of Li metal anode is in relationship with solvation shell structure of Li(solvent)*_x_* ^+^ cluster and Li^+^/Li^0^ diffusion across the solid interphase or electrode surface.^[^ ^13^, ^14^, ^15^ ^]^ However, as the operating temperature drops to low temperature below 0°C such as −20°C, the formation of dendrites becomes increasingly formidable owning to the sluggish kinetics of larger Li(solvents)*_x_* ^+^ shell evolution before electrolyte solidification, persistently degrading battery performances.^[^ ^16^, ^17^ ^]^

As known, the chemistry of Li(solvents)*_x_* ^+^ can be attributed to ion‐dipole interaction, which displays the central lithium ions and outer polar solvent molecules. And the strong ion‐dipole interaction triggers the specific orientation arrangement of solvent molecules, forming a tight solvation shell with a higher structural order and increasing the desolvation energy barrier significantly. Therefore, the process of removing solvation layer at low temperature requires overcoming higher energy due to the strongly enhanced ion‐dipole electrostatic interactions. Current strategies toward interfacial desolvation in LT‐LMBs mainly focus on electrolyte engineering through introducing diluter or addictive with high dielectric constant (ε) to weaken the ion‐dipole interactions,^[^ ^18^, ^19^, ^20^, ^21^, ^22^, ^23^ ^]^ therefore breaking down the sluggish Li^+^ solvation sheath. However, these regulation strategies are usually at the expense of sacrificing ion conductivity of electrolytes by expanding the solvent shell with co‐solvents or electrolyte additives.^[^ ^24^ ^]^ Meanwhile, porous metal‐organic frameworks (MOFs) enabled by modulating functional ligand (such as electron‐donor groups) for regulating the space charge distribution have already been extended to accelerate ion‐dipole interactions and separate the polar molecules.^[^ ^1^, ^25^, ^26^, ^27^, ^28^ ^]^ However, only some Li(solvents)*_x_* ^+^ groups can meet the pore size to pass through the pores to reach metallic Li surface. Decreasing the temperature, the large solvent cluster are hard to screen out owing to the increased strength of ion‐dipole interactions, retarding Li^+^ desolvation and diffusion kinetics. Regrettably, few studies are directly concentrated on promoting Li metal cyclability through breaking down interfacial ion‐dipole interactions at low temperatures.^[^ ^29^, ^30^, ^31^ ^]^

As accepted, the ion and electron behaviors at the inner Helmholtz plane (IHP) layer are always related to the electrolyte/electrode interface information.^[^ ^32^, ^33^, ^34^ ^]^ Alternatively, electrochemically catalyzing the ion‐dipole interactions would be the best choice for modulating the IHP layer to decrease desolvation barriers, where the Li^+^ transport would not be slowed down.^[^ ^13^, ^35^, ^36^ ^]^ Recently, to enhance catalytic sites and capability, electron delocalization engineering has been put forward to generate a higher density of free electron states for redistribution.^[^ ^37^, ^38^, ^39^, ^40^, ^41^, ^42^, ^43^ ^]^ In our former studies, the defect or atomic engineering by modulating *3d* or *4f* electron orbitals could break down the long‐range ordered bonds of crystal structure, contributing to more active sites and high catalytic efficiency.^[^ ^44^, ^45^, ^46^, ^47^, ^48^ ^]^ Therefore, screening highly active delocalization‐electron modulated metal‐based catalysts is desired toward the interfacial ion‐dipole interactions for fast Li^+^ desolvation and transport in the LT‐LMBs.

Herein, the emerging interfacial catalysis on regulating the ion‐dipole interactions to reconstruct Li(solvents)*_x_* ^+^ complexes under low‐temperature surrounding is proposed by electron delocalization engineering, uniformizing free ion flux and fast ion diffusion kinetics. In detail, a series of *d*‐orbital metal oxides (M = Ti, V, Fe, Co) are screened and systematically investigated at the electrode/electrolyte interface by the combination of density functional theory (DFT) simulation and electrochemical tests. With interface‐sensitive sum frequency generation (SFG) spectroscopy, in‐depth X‐ray photoelectron spectroscopy (XPS) and time‐of‐flight secondary‐ion‐mass‐spectrometry (TOF‐SIMS) and COMSOL simulation, the prototype delocalization‐electron modulated TiO_2‐x_ with adjustable electronic density anchored on carbon networks (DEM‐TO@C) performs the best behaviors against the ion‐dipole interactions between Li^+^ and solvent, thereby, accelerating desolvation kinetics of Li(solvents)*_x_* ^+^ complexes at low temperature. As a result, the optimized Li electrode delivers a dendrite‐free long lifespan of 1800 h at 1 mA cm^−2^ under −20°C. Even decreasing the temperature to ultralow −50°C, stable overpotential around 525 mV without any short‐circuit is achieved. The well‐matched ultrahigh loading Li‐LiNi_0.8_Co_0.1_Mn_0.1_O_2_ (NCM) cell (≈20 mg cm^−2^) and Li–S battery demonstrate excellent capacity retention nearly 100% and ultra‐steady rate capacity at shifting current rate under 0°C, signifying great promise for achieving practical high‐performance LT‐LMBs.

## 2 Results and Discussion

### 2.1 Simulations on Electron Structures and Ion‐Dipole Interactions

**Figure 1:**
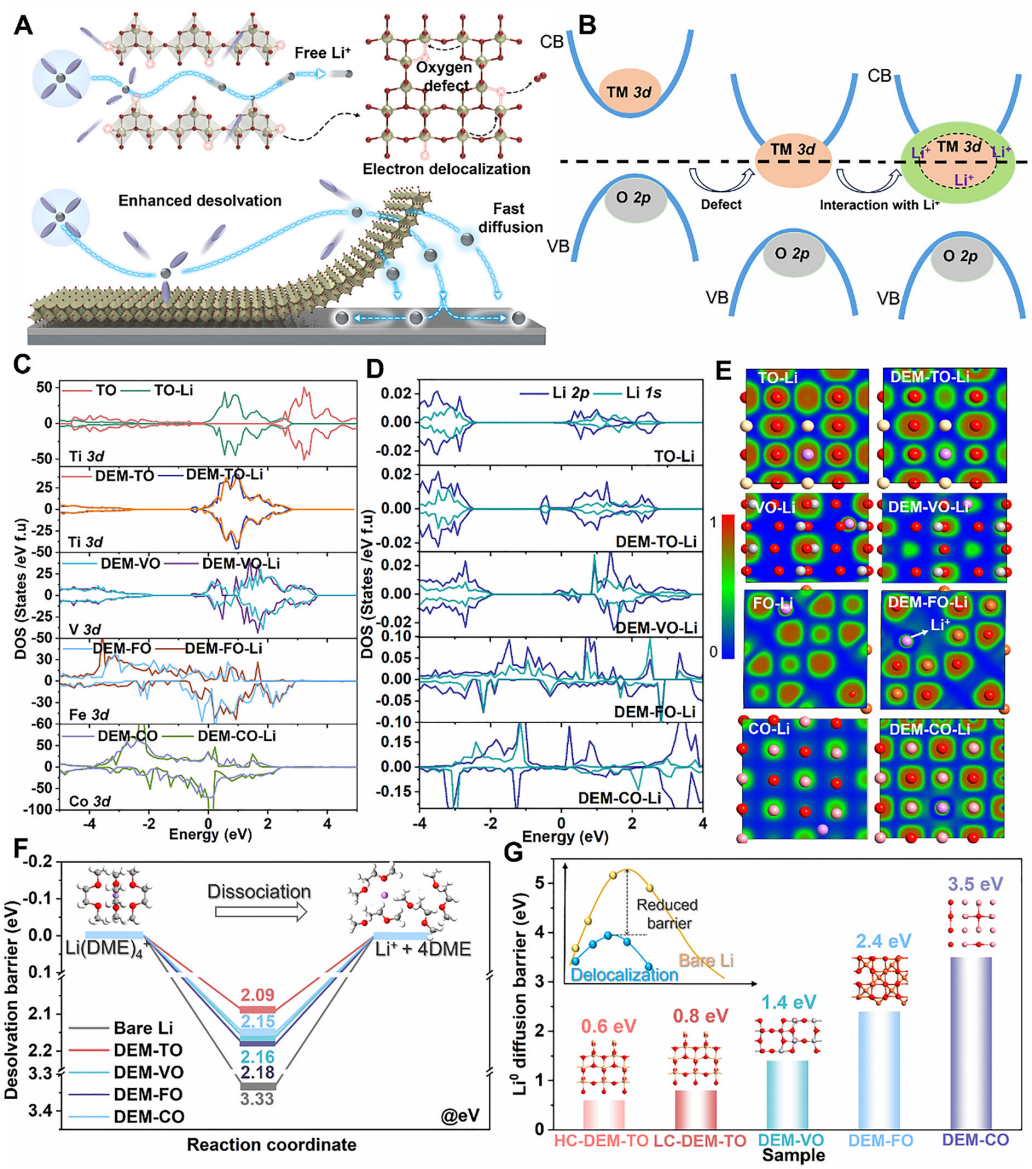
Simulation of electron delocalized catalysts on Li behaviors. A) Schematic illustration of the delocalization‐electron catalyzers on accelerating the Li^+^ desolvation and diffusion; B) Energy reconfiguration by defect engineering and the interaction with Li; C) The density of states (DOS) of Ti *3d*, V *3d*, Fe *3d*, Co *3d* with/without interaction of Li; D) The DOS of Li *1s* and Li *2p* from the surface atoms interacted with DEM‐TMOs; E) The 2D slice of charge density difference of Li^+^ species interacted with different DEM‐TMOs; F) Comparison of the desolvation energy barriers of Li(DME)_4_^+^ into Li^+^ and DME and (G) Diffusion energy barriers of Li atoms (Li^0^) on the different delocalization‐electron catalyzers.

The deep understanding of electronic engineering before and after interacting with Li atoms is beneficial for figuring out delocalized electron effects in kinetically propelling the ion‐dipole dissociation and Li ion/atom diffusion behaviors. As illustrated in **Figure** **1**A, the electron state density of delocalization‐electron modulated transition metal oxides (denoted as DEM‐TMOs) are expected to interact with Li(solvents)*_x_* ^+^ groups, providing affluent active sites to accelerate the initial ion‐dipole dissociation for Li(solvents)*_x_* ^+^ desolvation to release plentiful free Li^+^ for fast ion diffusion. In this regard, the typical *d*‐orbital metal oxides (including TiO_2_ (TO), V_2_O_5_ (VO), CoO (CO), and Fe_2_O_3_(FO)) are screened and investigated on the DEM‐TMOs with obvious shifted *3d*‐orbital after forming oxgen defects (Figure 1B). As desired, the conductivity band of *3d* metal in TO system is reduced and the bad gap shrinks by defect incorporation, and it turns back after interacting with Li^+^ (Figure S1, Supporting Information). Meanwhile, the structures and lattice parameter of TMOs with/without defects are displayed in Figures S2 and S3 (Supporting Information). Figure 1C and Figures S4 and S5 (Supporting Information) showcase that the evolution of density of states (DOS) moving toward the Fermi level in the DEM‐TMOs after Li interaction, and it evidences the adjustability and orbital hybridization of *3d* electron structure through oxygen defects, suggesting enhanced activity on DEM‐TMOs‐Li to facilitate charge transfer processes during electrochemical deposition. Due to the low concentration disturbance of O defects (Here is TiO_1.9375_), no obvious magnetic moment and structural distortion are observed, further confirming the delocalization electron effect instead of the reduction of Ti^4+^ to Ti^3+^ (Figures S6–S8, Supporting Information),^[^ ^49^ ^]^ which has also been verified by the following experimental results. Meanwhile, the high defects concentration makes it more prone to interact with electrons. Specifically, the interactions between the DEM‐TMOs and Li^+^ species attributing to the Li *1s* and Li *2p* orbitals electronic states are enhanced by electron hybridizations of *3d* orbitals, which are directly pushed to near or across the Fermi level (Figure 1D; Figure S9, Supporting Information). The electron transfer on the DEM‐TMOs is also reflected in the differential electron density (Figure 1E; Figure S10, Supporting Information). Obviously, strong charge interactions between DEM‐TMOs and Li are achieved in comparison to the TMOs and Li, indicating the electron hybridization between various metal *3d*‐orbitals and Li *1s* or Li *2p* orbitals of the Li^+^ species. The electronic states of the Li(DME)_4_^+^ interacted with DEM‐TMOs shift to the metal atoms surrounded by oxygen defects, while they are mainly centered around the Li^+^ core in the bare Li(DME)_4_^+^ system (Figure S11, Supporting Information). Once forming the electron redistribution, it prompts large electronic states around the Li^+^ core with the decreased barriers, indicating the weak ion‐dipole interactions.

As previously revealed, the Li(DME)*_x_* ^+^ possesses a higher desolvation barrier than the Li(DOL)*_x_* ^+^ analogue in the electrolyte, showcasing the stronger coordination ability of DME with the central Li^+^ than DOL.^[^ ^50^ ^]^ The Li^+^‐DME bond would be dramatically affected and weakened, proceeding the desolvation to generate free Li^+^ from the solvated ion‐dipole structures (Figure S12, Supporting Information). Encouragingly, the DEM‐TO overcomes the least desolvation energy barrier (2.09 eV) than the others (Ti<Co<V<Fe: 2.09<2.15<2.16<2.18 eV) (Figure 1F) in contrast to that of the bare Li (3.33 eV), exhibiting much lower desolvation energy barriers from Li(DME)_4_^+^ to free Li^+^ for the DEM‐TMOs catalyzers than the perfect TMOs ones (Figure S13, Supporting Information). Afterwards, metastable initial Li atoms (Li^0^) for subsequent nucleation and plating are evolved across the SEI layer. Under the help of electron delocalization, Li^0^ is likely to confront lower diffusion energy barriers than that of perfect TMOs (Figure 1G; Figures S14 and S15, Supporting Information), obeying the beneficial diffusion pathway. Among them, the DEM‐TO provides the lowest diffusion barrier (0.8 eV) for potential faster Li diffusion kinetics, and the greater level of electron delocalization (HC‐DEM‐TO) is more beneficial to decrease the Li^0^ diffusion energy barrier to as low as 0.6 eV (Figure 1G; Figure S16, Supporting Information). Thus, the Li^0^ diffusion energy with DEM‐TMOs obeys the sequences of Ti>V>Fe>Co (0.8>1.4>2.4>3.5 eV), exhibiting the potential for fast diffusion within low‐temperature batteries.

### 2.2 Characterizations of Electron Delocalization in Representative Catalyst

**Figure 2:**
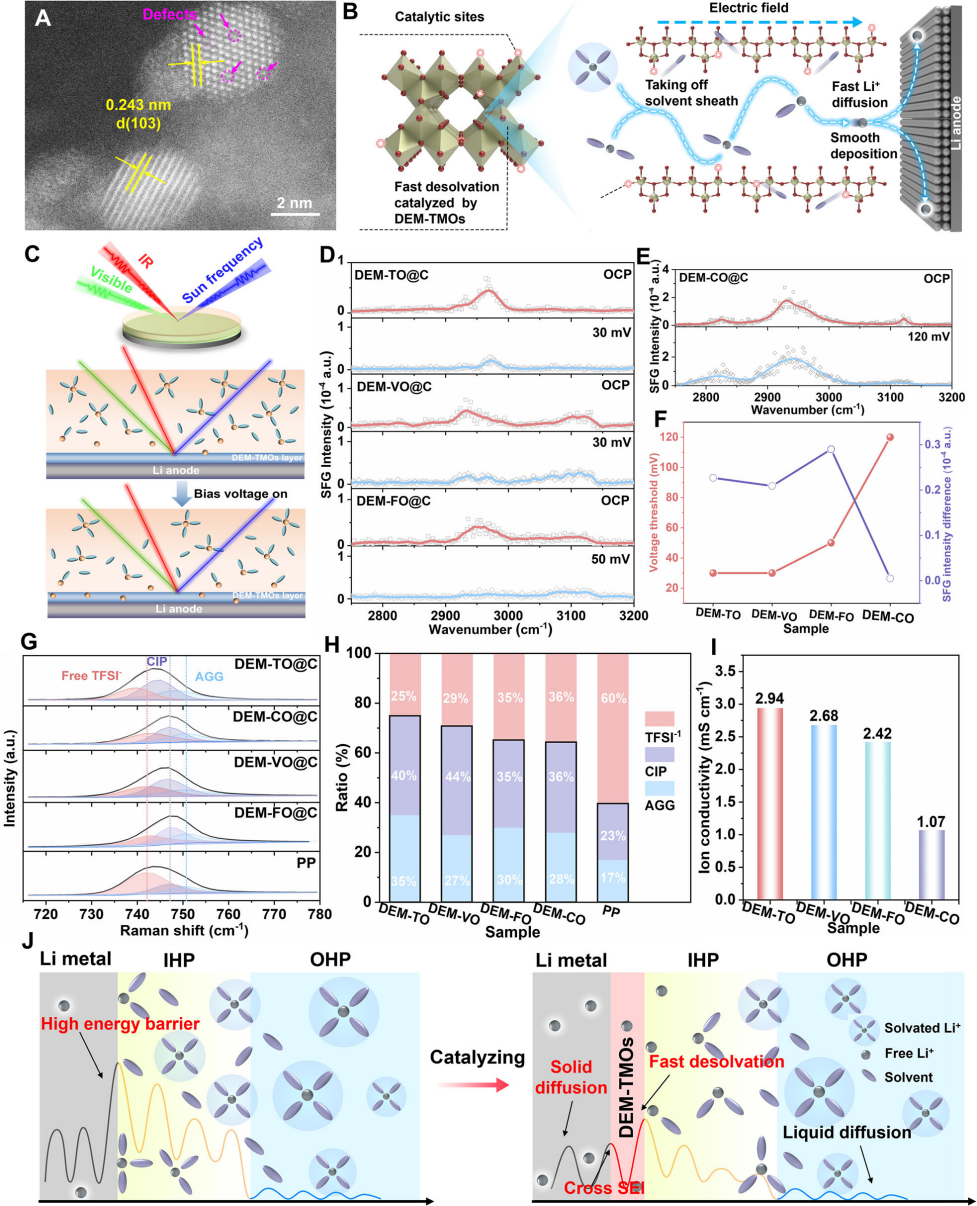
Desolvation investigation by in‐situ and ex‐situ Spectro‐electrochemical characterizations. A) HRTEM image of DEM‐TO@C; B) Schematic illustration of desolvation behaviors catalyzed by DEM‐TMOs@C catalyzers; C) Schematic illustration of the in‐situ SFG model with/without bias voltage; D‐E) The SFG spectra of solvated Li^+^ structure at the C–H region with/without DEM‐TMOs@C catalyzers under various bias voltages; (F) Comparison on the Li^+^ desolvation voltage threshold and the SFG intensity difference before and after desolvation; G) Comparisons of Raman spectra of the ether electrolyte with/without DEM‐TMOs@C catalyzers; H) The ratio summary of free TFSI^−^, CIP, and AGG in different systems; I) Comparison of Li^+^ conductivity with the DEM‐TMOs@C catalyzers; J) Schematic description of the reconstruction of IHP layer by DEM‐TMOs@C catalyzers.

To verify the electron delocalization effect, the representative DEM‐TO@C catalyst was firstly demonstrated (see details in the Supporting information). The crystal structure of DEM‐TO@C is verified by the Rietveld refinement, which is consistent with the crystalline structure of TiO_2_ (JCPSD No. 21–1272) without any phase changes or other heterogeneous phases (Figure S17, Supporting Information), but a slight redshift of E_g_ band in DEM‐TO@C is influenced by electron delocalization (Figure S18, Supporting Information). The scanning electron microscopy (SEM) images, scanning transmission electron microscopy (STEM) images and the energy‐dispersive X‐ray spectroscopy (EDX) mappings of the as‐synthesized DEM‐TO@C are shown in **Figure** **2**A and Figures S19–S22 (Supporting Information). All demonstrate that the DEM‐TO nanoparticles of ≈15 nm are uniformly dispersed in the typically cross‐linked network of the DEM‐TO@C with affluent assembled porous structure. In the high‐resolution STEM image (Figure 2A), the as‐formed oxygen defects are also recorded and witnessed, as highlighted by circles. The typical interplanar spacings of lattice fingerprint is estimated to be 0.243 nm, corresponding to d_103_ spacing of TiO_2_. A pronounced *g*‐factor near 2.003 in electron paramagnetic resonance (EPR) signal of the DEM‐TO@C (Figure S23, Supporting Information) also suggests a large proportion of electron redistribution.^[^ ^51^ ^]^ The increased peak intensity ratio (1.31 vs 0.96) in the high‐resolution O *1s* spectra reconfirms the successful formation of defect‐induced electron delocalization, and the slight shift of the Ti^4+^ 2P_2/3_ from 464.6 to 464.8 eV is observed in high‐resolution Ti *2p* spectrum (Figure S24, Supporting Information). Benefiting from the larger amount of oxygen defects, higher electron donor density (Nd) in the DEM‐TO@C is recorded in Mott−Schottky curves than that in TO@C (Figure S25, Supporting Information), which is attributed to the electron density reconstructed in the DEM‐TO@C.

### 2.3 Accelerated Ion‐Dipole Interaction Kinetics Visualized by In Situ Spectroscopies

As illustrated in Figure 2B, the ion‐dipole interactions in the larger Li(solvents)*_x_* ^+^ clusters are decomposed directly by the electrocatalytic effect of bridged DEM‐TMOs at the interface, allowing the released bare Li^+^ to participate in Li plating. Even decreasing to low temperature under 0°C, the DEM‐TMOs with intrinsic high electron/ion modulation capability also enables robust catalytic property to break the Li^+^‐DEM/DOL bonds and take off solvent molecules in the enlarged solvation shell structure. As known, the net desolvation process always takes place at the electrode/electrolyte interface rather than bulk electrolyte interior.^[^ ^52^ ^]^ To trace the desolvation evolution, the interface‐sensitive in‐situ SFG spectroscopy was applied to trace the evolution of desolvation (Figure 2C). As presented in Figure 2D–F, apparent C─H bond vibrations contributed by solvents in 2750–3200 cm^−1^ are recorded at the interfaces of DEM‐TMOs/electrolyte.^[^ ^44^ ^]^ Of note, a slight blue shift of the solvent peak emerges from open circuit potential (OCP) to bias voltage threshold (Figure 2D), which is attributed to electric‐field‐driven disruption of ion‐dipole coordination and corresponding structural reorganization within the Helmholtz layer. Upon applying various bias voltage onto the DEM‐TMOs/electrolyte systems, the interface is doomed to experience an initial solvent adsorption process until reaching the threshold voltage that starting desolvation procedure. Accordingly, the intensity of the C─H bond vibrations increases firstly and decrease suddenly with the advent of the threshold voltage (Figures S26–S28, Supporting Information). The initiation of Li^+^‐solvent dissociation exhibits voltage‐dependent selectivity due to the distinct desolvation energy barriers on different Li interface with/without DEM‐TMOs modulation layers. In other words, the dynamic process of dissociating the Li^+^‐solvents at the interface is initially started with different voltage threshold (Figure 2F; Figures S27 and S28, Supporting Information) in virtue of distinguished desolvation energy barriers. In comparison, the DEM‐TO exhibits moderate SFG intensity differences (≈2970 cm^−1^) with the lowest starting bias voltage for desolvation, showcasing the superior catalytic capability to the others, corresponding to the order of Ti≈V<Fe<Co (30≈30<50<120 mV), as summarized in Figure 2F.

The desolvation behavior was also witnessed by ex‐situ Raman spectra, the higher intensity ratio of CIP (one anion pairs with one Li^+^) and AGG (one anion pairs with two or more Li^+^) in the electrolyte or surface indicate the fast desolvation as it does in high concentration electrolyte. As displayed in Figure 2G, the fitting line and the deconvoluted peaks at approximately 742.6, 746.9 and 750.6 cm^−1^, assigning to free TFSI^−^, CIP and AGG species, respectively. In comparison, the DEM‐TO@C corroborates the highest total ratios (75%) of AGGs+CIPs, much higher than that of pristine electrolyte (40%) (Figure 2H), indicating the fastest desolvation behaviors at the catalytic interface. Consequently, the DEM‐TO@C acquires the fastest Li^+^ diffusion kinetics owing to the highest ionic conductivity across the bulk and interface among various catalysts (2.94>2.68 >2.42>1.07 mS cm^−1^) when using identical electrolyte volume (Figures 2I; Figure S29 and Table S1, Supporting Information). Beneficially, this electrochemical catalysis of the Li^+^‐solvents desolvation reaction would help to modulate the inner Helmholtz plane (IHP) layer with least energy consumption, which also breaks down the ion‐dipole interactions and changes interfacial desolvation‐diffusion behavior nearby the IHP layer of electronic double layer (EDL) (Figure 2J). Both the simulation and spectroscopical results have clearly demonstrated the improved kinetics under the great help of electron‐delocalization in DEM‐TMOs with marginally improved dielectric constant for affecting the ion‐dipole interactions, revealing delocalization‐electron modulated catalyzers are beneficial for rapid desolvation kinetics to release free Li^+^ at the electrode/electrolyte interface.

### 2.4 Performance of Li Plating Under Low Temperatures

**Figure 3:**
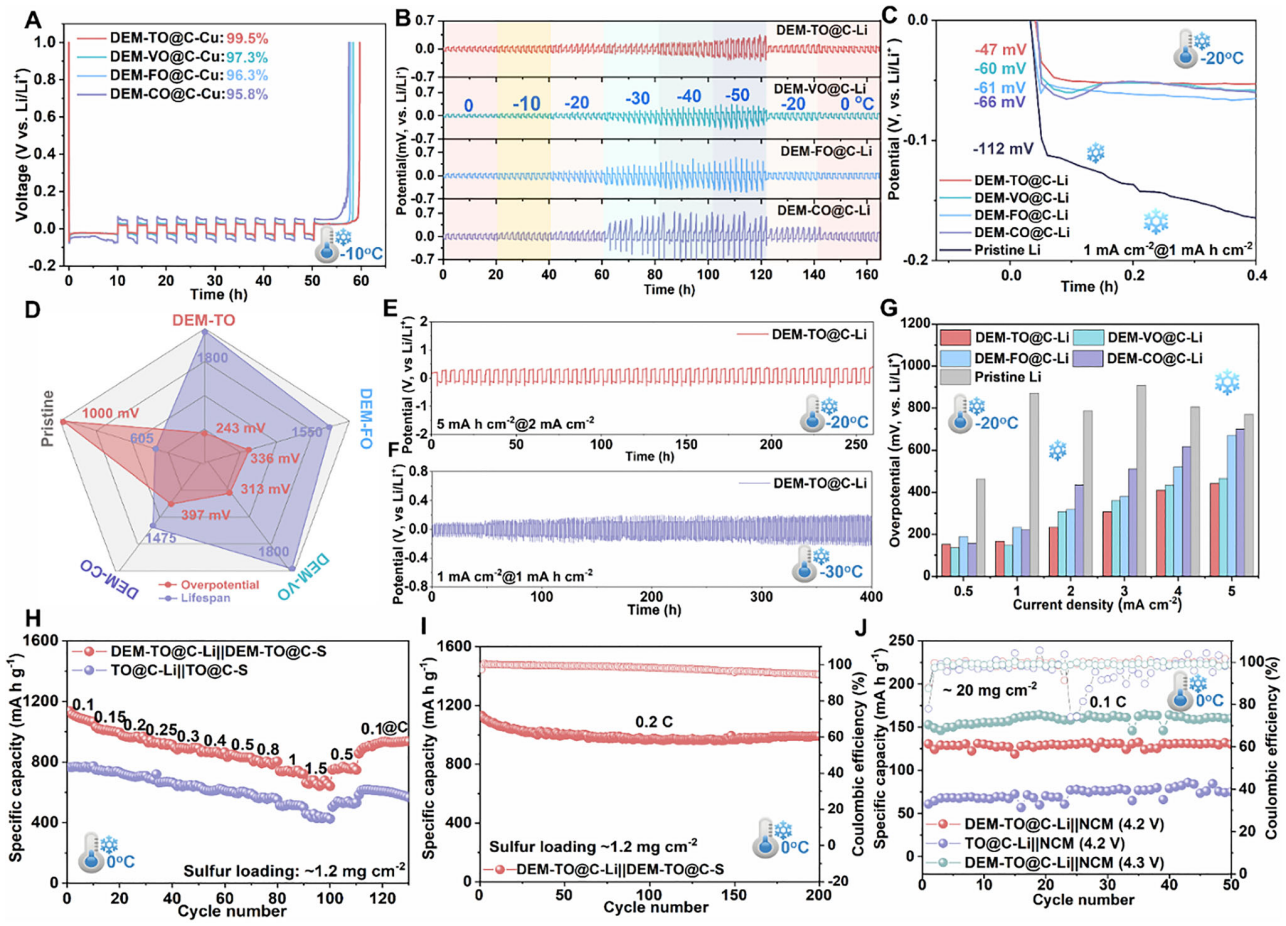
Low‐temperature performance with DEM‐TMOs@C on Li metal batteries. A) Aurbach CE test on asymmetrical cells; B) Constant galvanostatic plating/stripping stability from 0°C to −50°C at 1 mA cm^−2^; C) Comparison of initial Li nucleation on the DEM‐TMOs@C‐Li electrodes; D) Comparison of the plating/stripping lifespan and overpotential under −20°C; Galvanostatic plating/stripping stability of the prototype DEM‐TO@C‐Li electrode (E) under −20°C at 2 mA cm^−2^ with 5 mA h cm^−2^ and (F) under ‐30°C at 1 mA cm^−2^ with 1 mA h cm^−2^; G) Comparisons of overpotentials on DEM‐TMOs@C‐Li electrodes at different current densities; H) Rate performance of catalyzed full battery under 0°C; I) Cycling performance of the DEM‐TO@C catalyzed Li‐S full battery at 0.2 C under 0°C; J) Cycling performance of Li||NCM811 full batteries with ultra‐high loading of active materials charged to 4.2 V or 4.3 V under 0°C.

As known, under the low‐temperature surroundings above freezing point, the Li(solvents)*_x_* ^+^ becomes much larger, costing much higher energy to overcome desolvation.^[^ ^17^, ^18^ ^]^ As shown in Figures S30–S33 (Supporting Information), the electrochemical impedance spectroscopy (EIS) evolutions of symmetric cells with catalyzers display the increased charge transfer and mass transfer resistance in relation to the temperature decrease. Among them, the DEM‐TO@C modified Li (DEM‐TO@C‐Li) exhibits the smallest resistance among these cells (Figures S31 and Table S2, Supporting Information) even under ultralow temperature of −20°C. Also, the DEM‐TO@C‐Li displays the fastest Li ion transport kinetics of liberated Li^+^ compared with pristine Li (4.31 vs 331) (Figures S32 and S33, Supporting Information), keeping in consistence with the lowest energy barrier of Li^0^ diffusion simulated by DFT. Under ‐10°C, Li‐Cu asymmetric cells with/without *3d*‐orbital delocalized electron were compared (**Figure** **3**A), it shows a stable voltage profile and high Coulombic efficiency (CE) ranging from 95.8% to 99.5%. However, the pristine Li‐Cu or CNT‐decorated Cu only displayed obviously lower CE of merely 87.3% or 91.5%, respectively (Figure S34, Supporting Information). Furthermore, the overpotentials in Li‐Li cell with temperature decreasing from 0 to −50°C were investigated. As exhibited in Figure 3B, the overpotentials of the DEM‐TMOs@C‐Li electrode increase gradually whilst it almost maintains stable voltage profiles without any short‐circuit at each temperature. However, the DEM‐CO@C‐Li electrode presents an overpotential surge at ultralow −30°C and obvious short‐circuit with Li dendrite formation at ultralow −50°C, attributing to the sluggish interfacial Li^+^ desolvation and diffusion kinetics of the large volume impeded by the decreased operation temperature. Cycled at 1 mA cm^−2^ under −20°C, the initial nucleation barrier is reduced from 112 to 47 mV with the decoration of *3d*‐orbital regulated catalytic layers (Figure 3C; Figure S35, Supporting Information), suggesting the ≈2.5 times decrease of nucleation barrier. In the following cycling (Figure 3D; Figure S36, Supporting Information), all the Li electrodes deliver prolonged stripping/plating lifespans (1475–1800 h) with low overpotential (240–397 mV), which is better than pristine Li one (1000 mV). Specially, a stripping/plating lifespan up to 1800 h with ultra‐stable overpotential of 240 mV is achieved for the DEM‐TO@C‐Li. Instead, the pristine Li electrode only stabilizes for less than 400 h and then drops due to the dramatic potential surge (1000 mV). Even enhancing the current density and/or areal capacity (Figure 3E; Figure S37, Supporting Information), the DEM‐TO@C‐Li electrodes under −20°C still retain low overpotentials and stable lifespan of 250 h under 5 mA h cm^−2^. Further dropping temperature to −30°C, the DEM‐TO@C‐Li electrode can survive for 400 h (Figure 3F). Under −20°C, even reaching 5 mA cm^−2^, the DEM‐TO@C‐Li electrode outputs the fast diffusion kinetics and reversibility with the lowest overpotentials of merely 394 mV in comparison to 774 mV of pristine Li (Figure 3G; Figure S38, Supporting Information). Under the room temperature, the DEM‐TO@C‐Li electrodes do the same as under low‐temperature (Figure S39, Supporting Information). Overall, the DEM‐TO@C‐Li electrode behaves the best catalytic efficiency in desolvation and diffusion than the others, following the order of Ti>V>Fe>Co. Meanwhile, the cells based on C‐Li electrodes also displays poor performance under either low or room temperature surroundings, further confirming the catalytic effect of the DEM‐TMOs in dissociating the ion‐dipole interactions for fast kinetics (Figure S40, Supporting Information).

Based on the above screening results, the DEM‐TO@C‐Li electrode was coupled with sulfur cathode or LiNi_0.8_Co_0.1_Mn_0.1_O_2_ (NCM811) cathode. As expected, electron density reconstruction of catalyst enhances the chemisorption activity toward soluble sulfur species (Figure S41, Supporting Information), and a fast Li_2_S precipitation takes place on the DEM‐TO@C electrode (Figure S42, Supporting Information). Similarly, the fresh Li‐S full battery employed on DEM‐TO@C‐Li outputs much lower charge‐transfer resistance (61 vs 220 Ω) (Figure S43, Supporting Information), lower polarization (260 vs 410 mV) with better reversibility (Figure S44, Supporting Information), and higher lithium‐ion diffusion kinetics (8.07 vs 0.65) (Figure S45, Supporting Information), signifying better catalytic activity of DEM‐TO@C. The corresponding activation energy (E_a_) for Li^+^ transport is markedly descended (19.12 vs 22.86 kJ mol^−1^) (Figure S46, Supporting Information). Under 0°C, the delocalization‐electron catalyst modified Li‐S full battery signifies high reversible rate capacities of 1137 and 663 mA h g^−1^ at shifting current rate from 0.1 to 1.5 C (1C = 1675 mA g^−1^), respectively (Figure 3H; Figure S47, Supporting Information), which is much higher than the cell without electron delocalization (766 vs 457 mA h g^−1^). Meanwhile, the DEM‐TO@C modified full cell affords a capacity‐retention of 88% over 200 cycles at 0.2 C (Figure 3I; Figure S48, Supporting Information). Decreasing to −20°C, the cell with DEM‐TO@C‐Li electrode still delivers stable rate performance and cycling stability with high reversible capacity of 882 mA h g^−1^ after 150 cycles (Figure S49, Supporting Information). Moreover, the DEM‐TO@C‐Li electrode coupled with high‐loading LiNi_0.8_Co_0.1_Mn_0.1_O_2_ (NCM) (≈20 mg cm^−2^) electrode provides an excellent capacity retention of nearly ≈100% and ultra‐steady rate capacity at shifting current rate even under 0°C (Figure 3J; Figure S50, Supporting Information), which is comparable to or even better than the reported LMBs systems under low‐temperature surroundings (Table S3 and Figure S51, Supporting Information). Elevating the ambient temperature to room temperature, the DEM‐TO@C catalyzed Li‐S full cells still deliver reversible rate capacities of 613 mA h g^−1^ at 5 C (Figure S52, Supporting Information) and maintains the revisable capacity of 870 mA h g^−1^ after 200 cycles at 1 C (Figure S53, Supporting Information). A long‐term cycling lifespan of 1000 cycles at 3 C and even a fading rate of merely 0.036% per cycle at 5 C are achieved (Figure S54, Supporting Information).

### 2.5 Modulation Mechanisms of Catalysts on Ion‐Dipole Interactions

**Figure 4:**
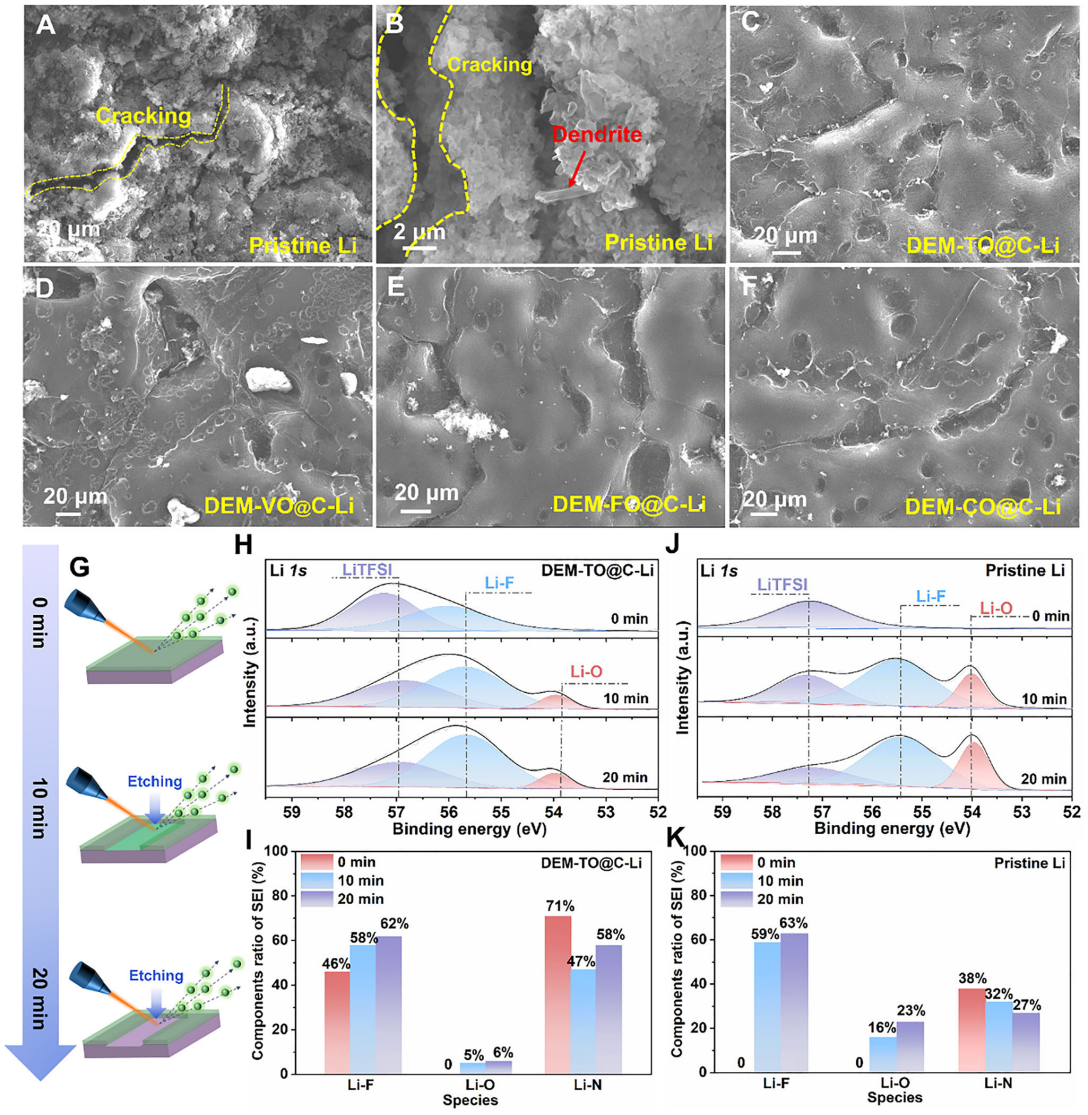
Surface characterization on cycled Li/electrolytes interface for underlying mechanism. The SEM images of (A,B) pristine Li electrodes, (C) DEM‐TO@C‐Li, (D) DEM‐VO@C‐Li, (E) DEM‐FO@C‐Li and (F) DEM‐CO@C‐Li from the Li||Li symmetric cells at the end of the cycling for 600 h under −20°C; G) Schematic illustration of the in depth XPS analysis probing at the Li/electrolytes interface; XPS depth profiles of SEI formed on the Li/electrolytes interface at the end of the cycling for 20 cycles at −20°C, including (H) high‐resolution Li *1s* spectra for DEM‐TO@C‐Li; I) Comparison of the component ratio of SEI layer on DEM‐TO@C‐Li electrode; J) High‐resolution Li *1s* spectra for pristine Li; K) Comparison of the component ratio of SEI layer on pristine Li electrode.

To directly observe the enhanced Li^0^ atom diffusion morphology with the DEM‐TMOs@C, the ex‐situ SEM images were recorded on the cycled Li metal surface after removing the catalytic layer (**Figure** **4**A–F; Figure S55 and S56, Supporting Information). Obvious cracks and needle like tips with ruptured dead lithium are shown on the pristine Li surface, and it triggers further growth of Li dendrite (Figure 4A,B). In contrast, the uniform but dense Li‐metal surface morphology directly confirms the effectiveness of the DEM‐TMOs@C in modulating initial ion‐dipole interactions for fast Li^+^ desolvation and Li^0^ diffusion to inhibit dendrite growth (Figure 4C–F; Figure S56, Supporting Information). It is found that the newly formed Li metal atoms are capable of penetrating through the catalytic layer and reaching the bottom metal surface to deposit smoothly. As a prototype, uniformity of Li plating with homogenous SEI was further detected by in‐depth XPS technology, as presented in Figure 4G–K and Figures S57 and S58 (Supporting Information). A prominent F–Li peak centered around 55.5 eV in the high‐resolution Li *1s* spectra (Figure 4H) and a strong Li–F peak close to 686.5 eV in the high‐resolution F *1s* spectra (Figure S57, Supporting Information) are observed, which is derived from the reduction of LiTFSI on Li metal surface. In detail, the content of the Li‐F species increases continuously from 46% to 62% throughout the etching from 0 to 20 min, while the C–F peak at 690 eV and the organic Li bond originated from LiTFSI species located around 57.3 eV decrease gradually from 25% to 0 and 54% to 31% (Figure 4I; Figure S57, Supporting Information), respectively. The reciprocal variation of these peaks fully proves the formation of Li‐F enriched SEI on the DEM‐TO@C regulated Li metal surface. Combining with the reduce of the Li–N species from 71% to 58% throughout the etching time in the N *1s* spectrum (Figure 4I; Figure S57, Supporting Information), it indicates that SEI layer also contains the Li_3_N. In a strong comparison, much lower ratio of Li‐N species and irregularly distributed F–Li species are collected on pristine Li surface, signifying the unevenness and cracking of the formed SEI at the top of the pristine Li surface (Figure 4J–K; Figure S58, Supporting Information).

**Figure 5:**
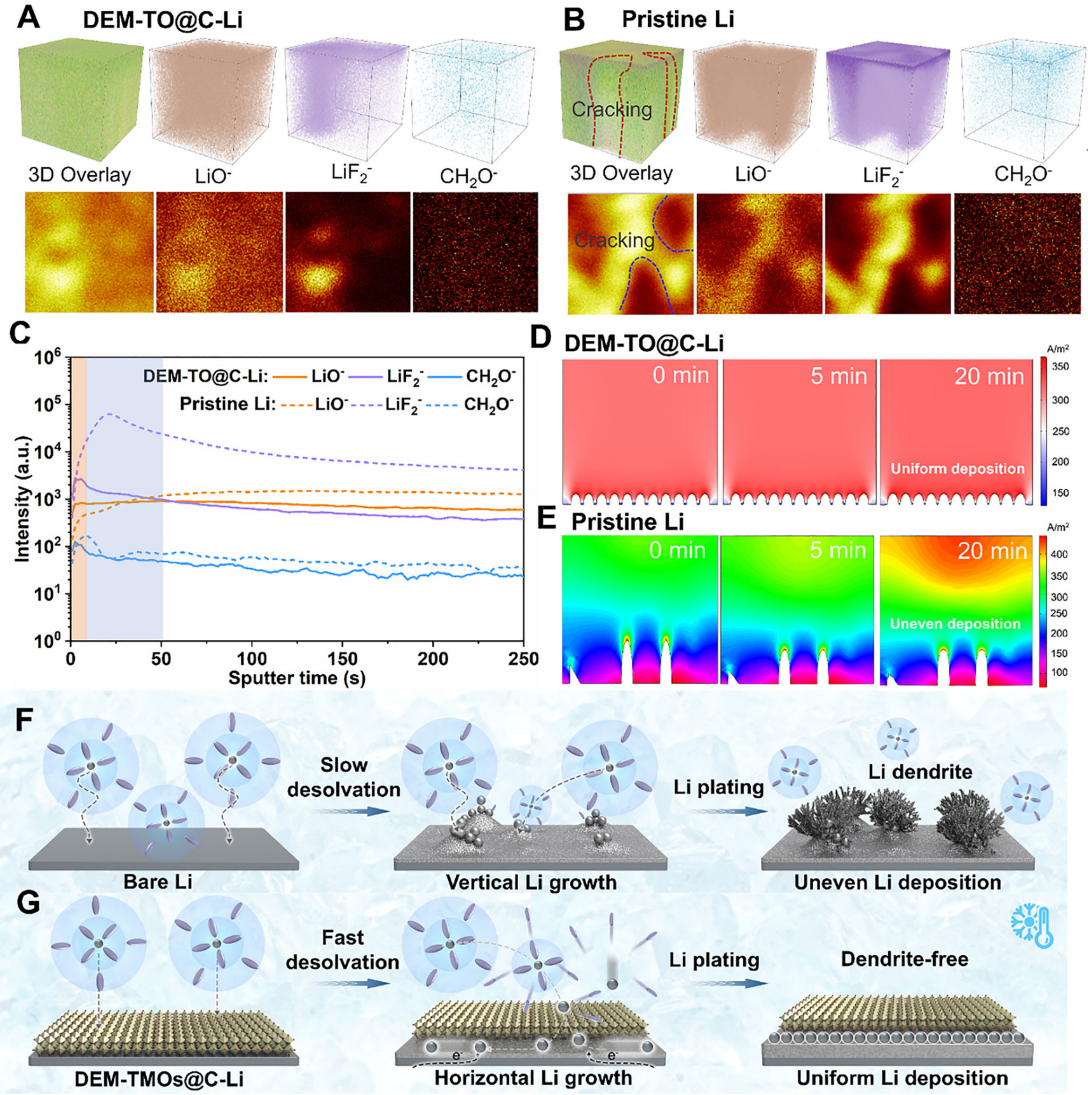
Mechanism illustration of DEM‐TMOs@C catalyzers in regulating lithium plating behaviors. Interfacial 3D and 2D reconstruction of organic/inorganic species within interfacial SEI on (A) DEM‐TO@C‐Li and (B) pristine Li after cycling for 20 cycles at −20°C, respectively; C) Intensity comparison of organic/inorganic species within interfacial SEI on cycled DEM‐TO@C modified Li and pristine Li electrodes; COMSOL simulation of the Li^+^ flux distributions on (D) the DEM‐TO@C‐Li and (E) pristine Li electrodes from a side view, respectively; Schematic illustration of (F) dendritic lithium plating behaviors on pristine Li and (G) DEM‐TO@C‐Li experiencing propelled desolvation and atom diffusion for lateral deposition under low‐temperature.

The 3D morphology and species distribution are further determined by time‐of‐flight secondary‐ion‐mass‐spectrometry (TOF‐SIMS) on Li metal anode after plating/stripping for 20 cycles at 1 mA cm^−2^ with 1 mA h cm^−2^ under −20°C. A bulk layer of well‐distributed Li with smooth and uniform SEI layer are observed for the cycled DEM‐TMOs@C‐Li electrodes (**Figure** **5**A; Figures S59–S61, Supporting Information), in comparison to the apparent rupture Li layer with rough plating surface in the cycled pristine Li (Figure 5B; Figure S60, Supporting Information). Under ion sputtering, the Li‐related species (such as LiF_2_^−^) along with the organic species (CH_2_O^−^) on the surface increase firstly and later decrease to constant as the increase of sputter time (Figure 5C; Figure S61, Supporting Information), reflecting the change of the organic and inorganic SEI components. More importantly, the longer durations of the changes for pristine Li than that for DEM‐TMOs@C regulated Li suggest the thicker SEI layer at the surface of cycled pristine Li (Figure 5C; Figure S62, Supporting Information). As expected, the introduction of DEM‐TMOs@C catalytic layers is beneficial to reconstruct the IHP layer and promote the formation of a robust and thin F, N‐rich SEI film on the surface of Li metal. Figure 5D,E depict the Li plating behaviors with/without DEM‐TO@C layer. A homogeneous distribution of Li^+^ with negligible concentration difference at the electrolyte and electrode interface during the electroplating process due to the homogenized and reduced local electric/concentration field under DEM‐TMOs modulation (Figure 5D). However, “hot spots” corresponding to significantly enhanced electric/concentration fields can be observed on the unmodified surfaces of Li foil (Figure 5E; Figure S63, Supporting Information), self‐amplifying Li dendrite formation.^[^ ^53^, ^54^ ^]^

Based on the above theoretical simulation and experimental findings, the functions of DEM‐TMOs@C in stimulating solvated Li^+^ desolvation and Li^0^ diffusion under low‐temperature are proposed (Figure 5F–G). Under the low‐temperature surrounding, in comparison with pristine Li, the partial dissociated Li(solvents)*_x_* ^+^ tends to co‐deposit to form loose and nonuniform distribution, inducing the formation and growth of Li dendrites in the following plating (Figure 5F). However, the solvated Li^+^ structure is immediately dissociated to generate isolated Li^+^ once contacting with the delocalization‐electron modulated catalytic layer due to the remarkably weakened ion‐dipole interactions, which has been fully elaborated on above DFT calculations, electrochemical tests and in‐situ/ex‐situ spectroscopical measurements. The free Li^+^ flux is further preferentially guided by the DEM‐TMOs@C catalytic layer to diffuses horizontally along the DEM‐TMOs@C modulation layer after coupling with electrons (Figure 5G), rendering a superior Li utilization and long lifespan.

## 3 Conclusion

In summary, a new concept of electron delocalization engineering to enhance catalytic efficiency is demonstrated to accelerate the Li(solvent)*_x_* ^+^ desolvation kinetics and diffusion kinetics of Li species under low temperature surroundings by regulating the ion‐dipole interactions. As a proof‐of‐concept, various delocalization‐electron modulated catalyzers (DEM‐TMOs@C) on Li behaviors are screened from theoretical simulations to experimental details. Among them, the prototype DEM‐TO@C exhibits the best catalytic effect on dissociation of ion‐dipole interactions within Li(solvent)*_x_* ^+^ groups and Li^0^ diffusion than the others (Ti>V>Fe>Co). Consequently, the optimal Li electrode contributes to a dendrite‐free behavior under the temperature as low as −50 °C and long lifespan up to 1800 h without any short‐circuit under −20 °C. The paired lithium sulfur (Li‐S) full battery enables a high‐capacity retention of 88% at 0.2 C over 200 cycles under 0°C. Moreover, the practical Li‐NCM cells with ultra‐high loading up to ≈20 mg cm^−2^ demonstrate an excellent capacity retention of ≈100% and stable rate performance under 0°C, highlighting the potential application of LMBs in harsh low‐temperature surroundings.

## Conflict of Interest

The authors declare no conflict of interest.

## Supporting information



Supporting Information

## Data Availability

The data that support the findings of this study are available from the corresponding author upon reasonable request.
