# Supplementary material for: Taming Interfacial Ion‐Dipole Interactions With d‐Orbital Delocalized Electron Catalysis Expediates Low‐Temperature Li Metal Batteries
Source: Adv Mater. 2025 Oct 11;38(4):e10894. doi: 10.1002/adma.202510894 (PMC12810596; doi:10.1002/adma.202510894)
Supplement: Supplementary file 1 — Supporting Information [file ADMA-38-e10894-s001.docx]

**Supporting Information**

**Taming Interfacial Ion-Dipole Interactions with d-Orbital Delocalized Electron Catalysis Expediates Low-temperature Li Metal Batteries**

*Jing Zhang, Fangqi Liu, Rong He, Qinghua Guan, Na Tian*, Jian Wu, Zhenjiang Cao, Shikai Yin, Yongzheng Zhang, Lujie Jia, Xifei Li, Caiyin You, Haitao Liu, Meinan Liu, Yidong Miao, Hongzhen Lin*, & Jian Wang**

J. Zhang，R. He, N. Tian, S. Yin, X. Li, C. You

School of Materials Science and Engineering, Xi’an University of Technology, Xi’an 710048, China

E-mail: tianna@xaut.edu.cn

Q. Guan, L. Jia, M. Liu, H. Lin

*i*-Lab & CAS Key Laboratory of Nanophotonic Materials and Devices, Suzhou Institute of Nano-tech and Nano-bionics, Chinese Academy of Sciences, Suzhou 215123, China

E-mail: hzlin2010@sinano.ac.cn

J. Wang

Helmholtz Institute Ulm (HIU), Ulm D89081, Germany

Karlsruhe Institute of Technology (KIT), Karlsruhe D76021, Germany

E-mail: jian.wang@kit.edu; wangjian2014@sinano.ac.cn

F. Liu, J. Wu

College of Advanced Interdisciplinary Studies, National University of Defense Technology, Changsha 410073, China

Y. Zhang

School of Textile & Clothing, Nantong University, Nantong, 226019, China

Z. Cao

School of Chemistry, Engineering Research Center of Energy Storage Materials and Devices, Xi’an Jiaotong University, Xi'an, Shaanxi 710049, China

H. Liu

Institute of Applied Physics and Computational Mathematics, Beijing 100088, China

Y. Miao

School of Materials and Chemical Engineering, Xuzhou University of Technology, Xuzhou, 221018, China

**Experimental sections**

*Synthesis of DEM-TMOs@C nanocomposites*

DEM-TO@C was prepared via a solvothermal method followed by a hydrogen thermal reduction method. In detail, the purified multi-walled carbon nanotubes (CNTs) powder (200 mg) was dispersed into 140 mL absolute ethyl alcohol under sonication to form homogeneous suspension. Butyl titanate (0.6 mL) was then added dropwise to the suspension with continuous stirring for 1 h. After that, about 3 mL ammonium hydroxide and 3 mL deionized water were dropwise added into the former mixture under vigorous stirring for 30 min. Subsequently, the mixture was transferred into a Teflon-lined stainless-steel autoclave and heated to 180 °C for 5 h. After cooling to room temperature, the resultant solid product was collected by filtration, rinsed by deionized water and ethanol thoroughly in sequence, and later freezing dried for 24 h. Thereafter, the DEM-TO@C nanocomposites were obtained by calcining the precursor at 450°C for 2 h under an Ar/H_2_ (5%:95%, by volume) mixture atmosphere, with a heating rate of 5 ^o^C min^-1^. The other DEM-TMOs@C were also synthesized using the similar method of combining solvothermal method with hydrogen thermal reduction.

*Preparation of sulfur cathodes nanocomposites*

The sulfur cathodes nanocomposites were prepared through in-situ liquid reaction method. Typically, the above synthesized DEM-TO@C (200 mg) was suspended in 300 mL deionized water under sonication, then Na_2_S*x* solution (1 mol L^-1^) was added dropwise into the suspension under continuous stirring. Afterwards, 2 mol L^-1^ HCOOH solutions was dripped slowly to the uniform blend for in-situ loading of sulfur on the DEM-TO@C matrix with continuous stirring overnight. The resultant composites were vacuum filtrated, rinsed repeatedly and freezing dried for 24 h. Finally, the dried collection was sealed in a vessel full of argon gas and heated at 155°C for 12 h to efficiently infiltrate sulfur into the porous matrix to generate DEM-TO@C@S composites.

*Assembly of symmetric and asymmetric coin cells*

Before assembling, the synthesized DEM-TMOs@C nanocomposite (10 mg) with surfactant was ultra-sonicated in ethanol (20 mL) to form homogeneous dispersion. Afterwards, the artificial catalytic layer was coated by vacuum-filtrating on the commercial separator (Celgard 2350) with an areal loading of 0.407 mg cm^-2^.

The electrochemical measurements were all conducted on 2025 type coin cells in a glove box full of pure Ar atmosphere. In the Li||Cu asymmetric coin cells (2025), the as-prepared DEM-TMOs@C catalytic layer was assembled covering the working electrodes (Li and Cu foil), and the coupled Li foil modified using the same method was employed as counter electrode. In the Li||Li symmetric coin cell, the as-prepared DEM-TMOs@C catalytic layer was assembled facing the commercial Li foils. About 80 μL electrolyte (1 mol L^-1^ LiTFSI dissolved in a mixed solvent of DME/DOL in volume ratio of 1:1) was dropped into each Li||Li and Li||Cu cell.

To assemble the catalysis related symmetric cell, 0.2 mol L^-1^ homogeneous Li_2_S_6_ solution is firstly synthesized through dissolving Li_2_S and sulfur in 1 mol L^-1^ lithium bis(ﬂuorosulfonyl)imide (LiTFSI) in solvent of 1,2-dimethoxyethane (DME) and 1,3-dioxolane (DOL) in volume ratio of 1:1 under continuous stirring for 24 h at 60 ^o^C. And the symmetric cell was set up using DEM-TO@C electrode as the cathode/anode separated by Celgard 2350 separator with 40 µL of above prepared polysulfide electrolyte, on which the liquid-solid conversion kinetics through Li_2_S precipitation analysis was measured.

*Li-S/NCM full cells assembly*

The cathode powder was prepared by mixing the as-synthesized sulfur nanocomposites or NCM, carbon black, and polyvinylidene fluoride (PVDF) as binder in an appropriate amount of N-methyl-pyrrolidinone (NMP) solvent with a weight ratio of 7:2:1 through continuous stirring till forming uniform slurry. Then, the fully mixed slurry was coated on aluminum foil to form an even layer by a doctor blade, which was followed by drying at 60 °C in a vacuum oven for 48 h. The working electrode was punched into discs of 11 mm in diameter with average loading of ~1.2 mg cm^-2^ and ~20 mg cm^-2^. The as-designed integrated full cells were assembled with DEM-TO@C@S or NCM cathode, and modified DEM-TO@C-Li anode separated by Celgard 2350 commercial separator, dropping the electrolyte (1 M LiTFSI with 1 wt% LiNO_3_ dissolved in mixed solvent of DME/DOL in volume ratio of 1:1).

**Materials and device characterizations**

The morphology of materials and electrodes were collaboratively characterized by scanning electron microscopy (SEM) on a Germany MERLIN compact scanning electron microscope (Zeiss Sigma HD) and transmission electron microscopy (TEM) on a spherical aberration corrected scanning transmission electron microscope (JEM-ARM200CF NEOARM) with an energy dispersive spectrometer, respectively. The X-ray diffraction (XRD) patterns for material structure characterization were collected on an XRD-7000S X-ray diffractometer using Cu Kα radiation in a 2θ range from 10° to 80°. To determine the defective structure, the Raman spectra were collected on a Horiba LabRAM ARAMIS spectrometer. The electron paramagnetic resonance (EPR) was measured on JEOL FA200. The X-ray photoelectron spectra (XPS) were collected on an ESCALAB 250XI system to investigate the change in the valance band and electronic structure of the nanocomposites. The XPS depth profiling was performed by sputtering Ar ions and the interfacial information was recorded at etching time of 0 s, 600 s and 1200 s. The sum frequency generation (SFG) is a commercial device using a copropagating configuration. With the picosecond laser system, the visible green light wavelength is fixed at 532 nm while the IR pulse is adjustable from 1000 to 4000 cm^-1^. For SFG measurements, the two lights directly shine on the electrode/solvent interface. And then the sum frequency light is generated and reflected to the detector. The 3D TOF-SIMS structures of the electrodes were performed on IONTOF.

The galvanostatic charge-discharge tests of the Li||Cu asymmetric batteries, Li||Li symmetric batteries and Li-S/NCM full batteries under different current rates were conducted on a Neware Battery Testing System (BTS-5 V 20 mA). Electrochemical impedance spectroscopy (EIS) and cyclic voltammetry (CV) of coin cells were performed with a VMP-3 electrochemical working station. The EIS plots were collected within the frequency range from 100 kHz to 0.01 Hz. For the *ex situ* SEM measurement, the Li electrodes were disassembled from coin cells in a glove box and then were washed with dimethyl ether and dried in vacuum overnight before further SEM characterizations.

**Theoretical Simulation Methods**

First-principles calculations are performed based on spin-polarized density functional theory (DFT) as implemented in the Vienna ab initio simulation package (VASP)^1,2^ and Quantum ATK^3^. The exchange-correlation potential is explained by the Perdew-Burke-Ernzerhof (PBE) functional based on spin-generalized gradient approximation plus on-site Coulomb interaction (SGGA + U) functional ^4,5^ with U_eff_ = 5 eV on M-3d (Ti, V, Fe, Co)^6^. The projector augmented-wave method is used for wave function expansion with an energy cutoff of 450 eV. The geometry optimization continues until the energy differences and ionic forces converge to less than 10^-6^ eV and 0.01eV/Å, respectively. Monkhorst-Pack k-point meshes of 10 × 10 × 1 are used for electronic structure calculation of metal oxides (including oxygen atom defects and Li ion adsorption). Calculation of desolvation energy barriers of Li (DME)_4_^+^ into Li^+^ and DME on the different delocalization-electron catalyzers is set k-point meshes of 5 × 5 × 1. To better describe the unbound interlayer interactions between Li(DME)*_x_*^+^ and DEM-TMOs, a semi-empirical dispersion potential (D) was added to the conventional Kohn-Sham DFT energy via Grimme's DFT D2 approach for the dyadic force field. At the same time, the calculation details of the desolvation energy here are described as follows: in the blank electrolyte, the completed dissociation of Li (DME)₄⁺ was calculated based on the following formula: E_a_=E(Li^+^) + E(4DME) -E(Li(DME)₄⁺), where the E(Li(DME)₄⁺), E(_Li_^+^) and E(DME) respectively represent the system energy of Li (DME)_4_^+^, Li ion, and 4DME. Subsequently, we also calculate the energy of Li (DME)₄⁺ after considering the effect of DEM-TMOs on ion-dipole interactions and the energy was calculated based on the following formula: E_a_=E(Li^+^@DEM-TMOs) + E(4DME@DEM-TMOs) - E(Li(DME)₄⁺@DEM-TMOs). For example, the dissociation barrier in the DEM-TO system is calculated and based on E_a_=E(Li^+^@DEM-TO) + E(4DME@DEM-TO) - E(Li(DME)₄⁺@DEM-TO)=2.09 eV.

The charge density difference (CDD) is calculated to qualitatively describe the charge transfer between the Li and metal oxides, which is defined as follows:

$$\Delta\rho=\rho_{Li@MO}-\rho_{Li}-\rho_{MO}$$

Where $\rho_{Li@MO}$ is the charge density of the system of lithium-ion adsorption on metal oxide surfaces, $\rho_{Li}$, $\rho_{Mo}$ are the charge densities of lithium-ion and metal oxides, respectively.


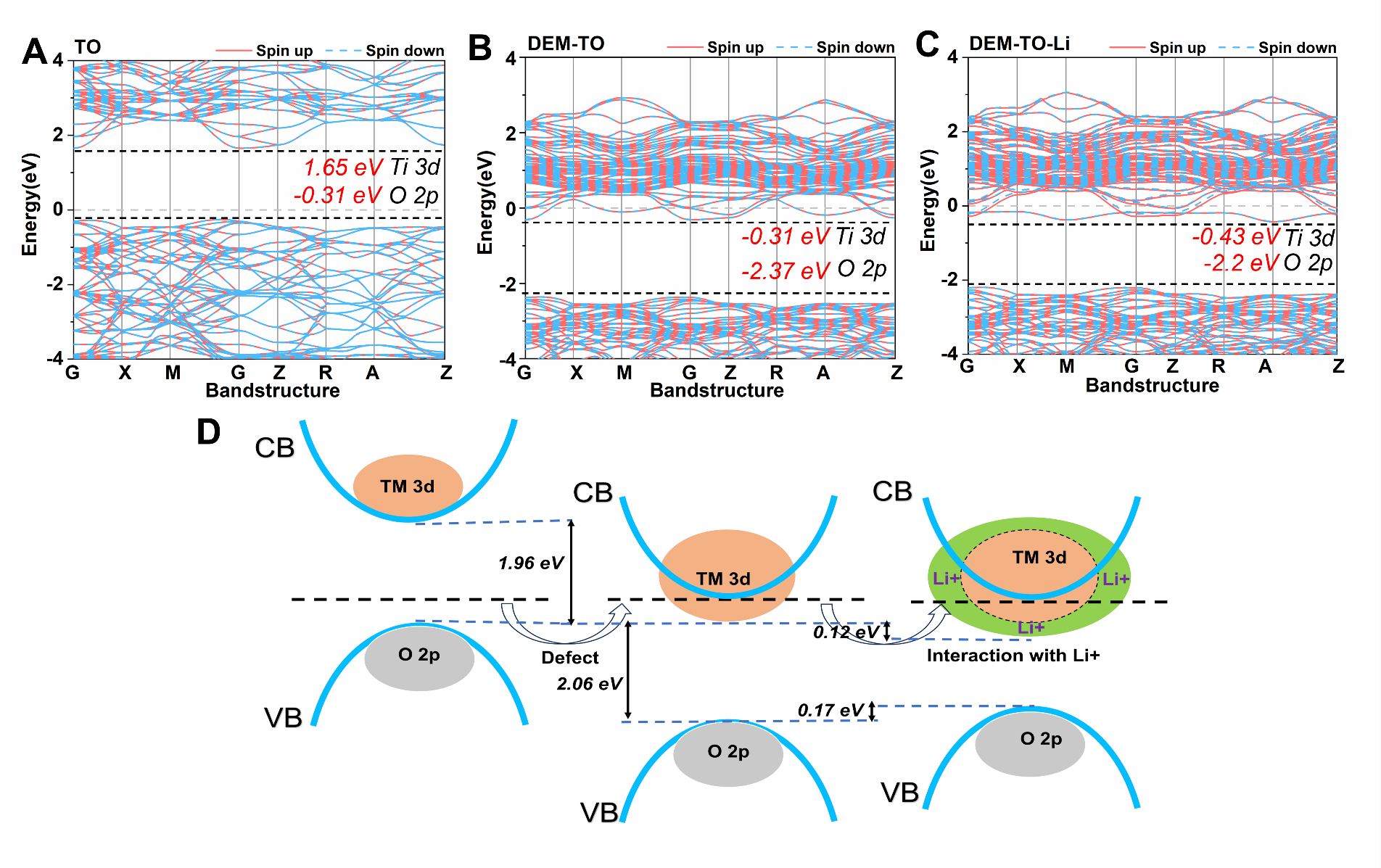


**Figure S1.** (A-C) The band structure evolution of TO after introducing different oxygen defect concentration; (D) Graphical illustration of energy reconfiguration for DEM-TO before and after the interaction with Li.


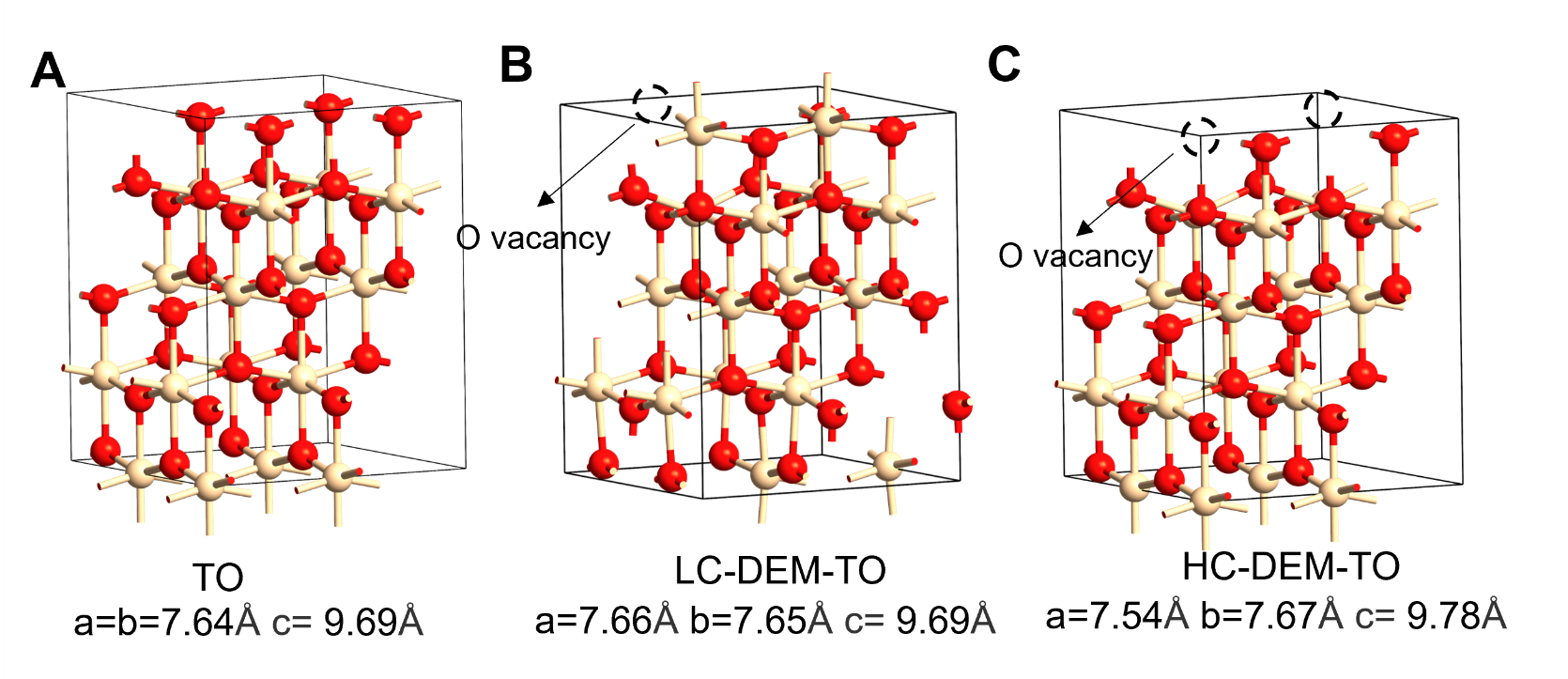


**Figure S2.** The crystal cell structures of TO and DEM-TOs and lattice parameter changes under different oxygen defect concentration.


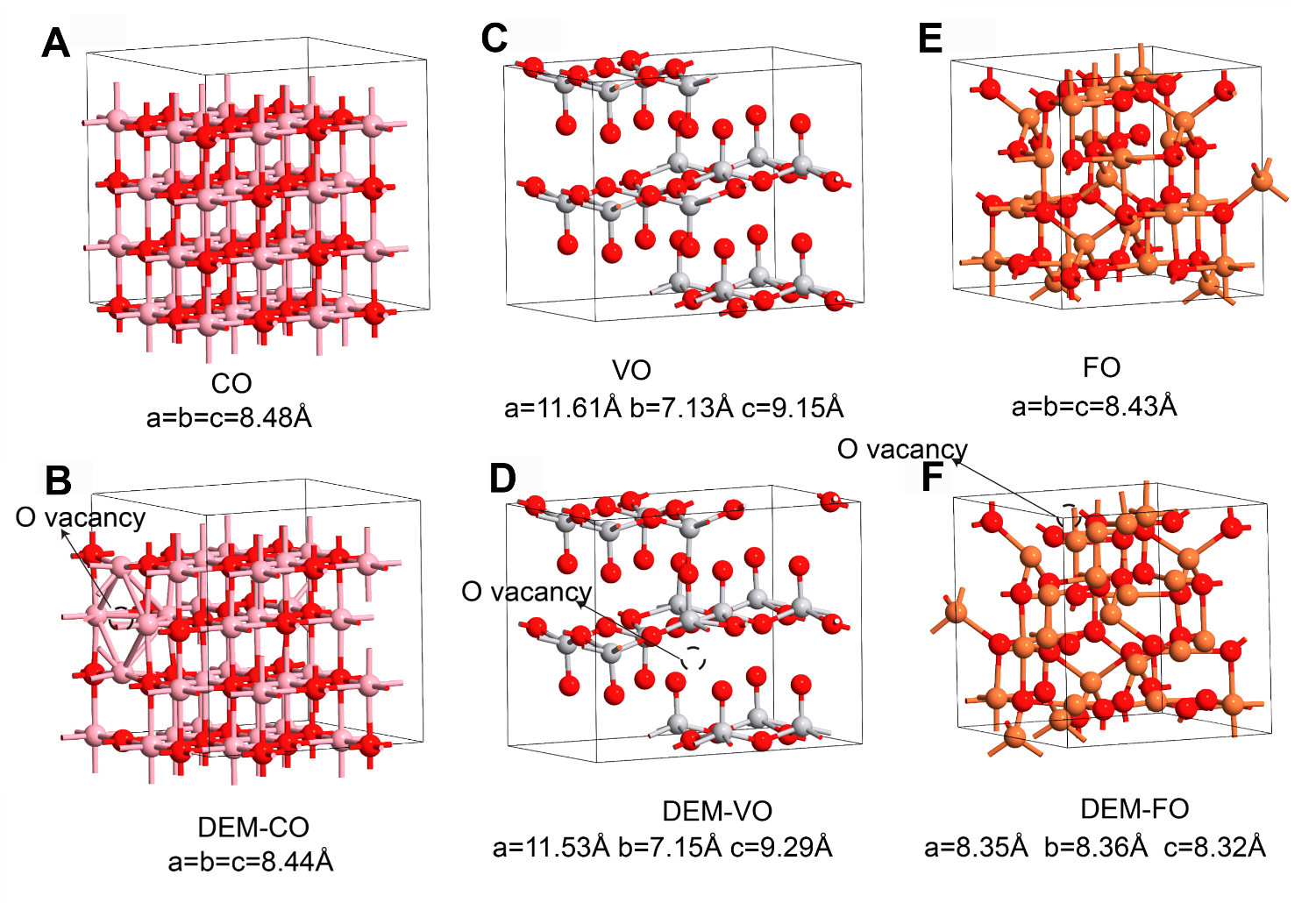


**Figure S3.** The crystal cell structures of TMOs and DEM-TMOs along with the lattice parameter changes.


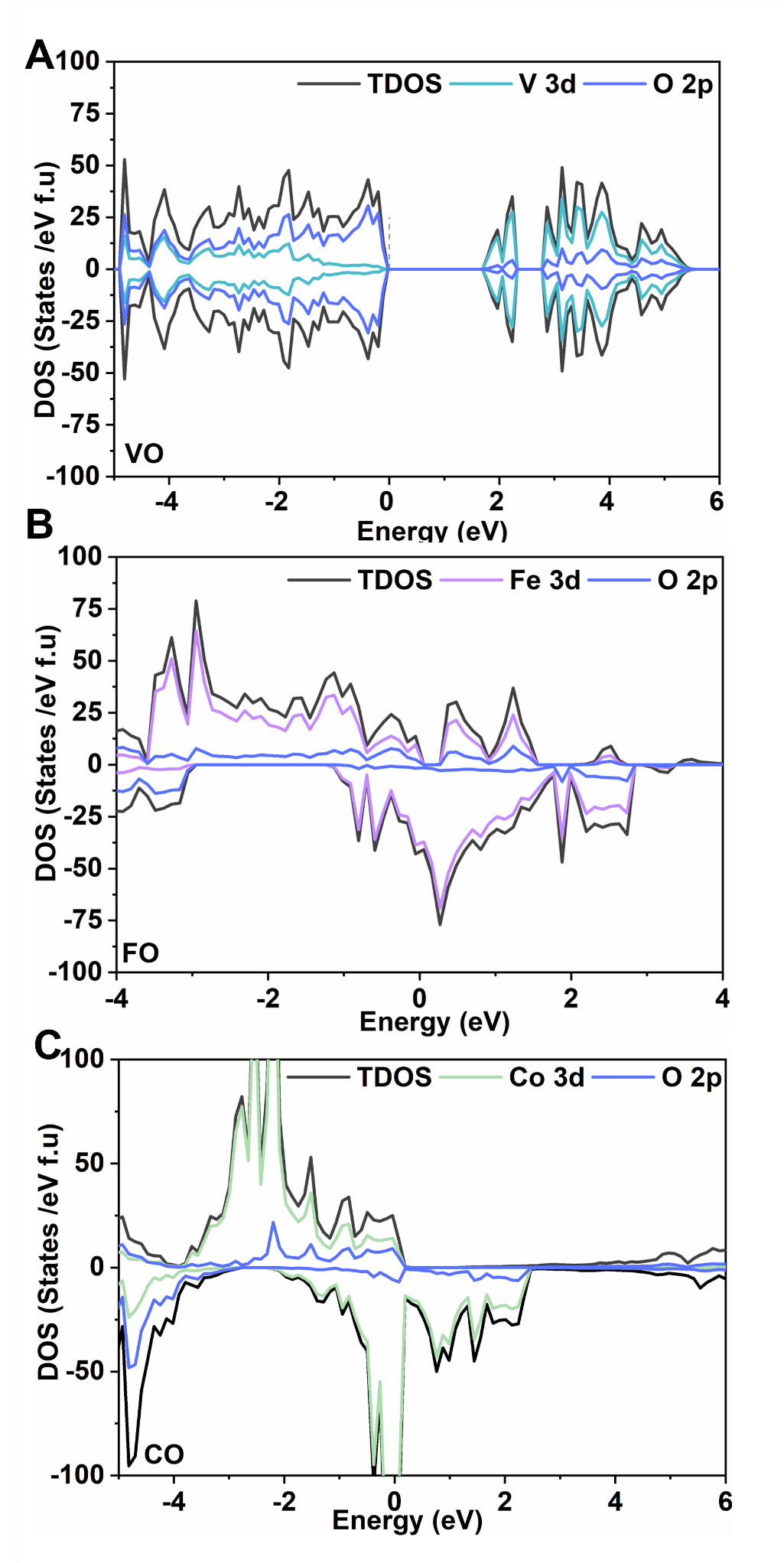


**Figure S4.** The density of states (DOS) of V *3d*, Fe *3d*, Co *3d* and O *2p* simulated from the surface atoms configurations in *d*-orbital metal oxides (M= V, Fe, Co).





**Figure S5.** The density of states (DOS) of O *2p* simulated from the surface atoms configurations in *d*-orbital metal oxides (M= Ti, V, Fe, Co).


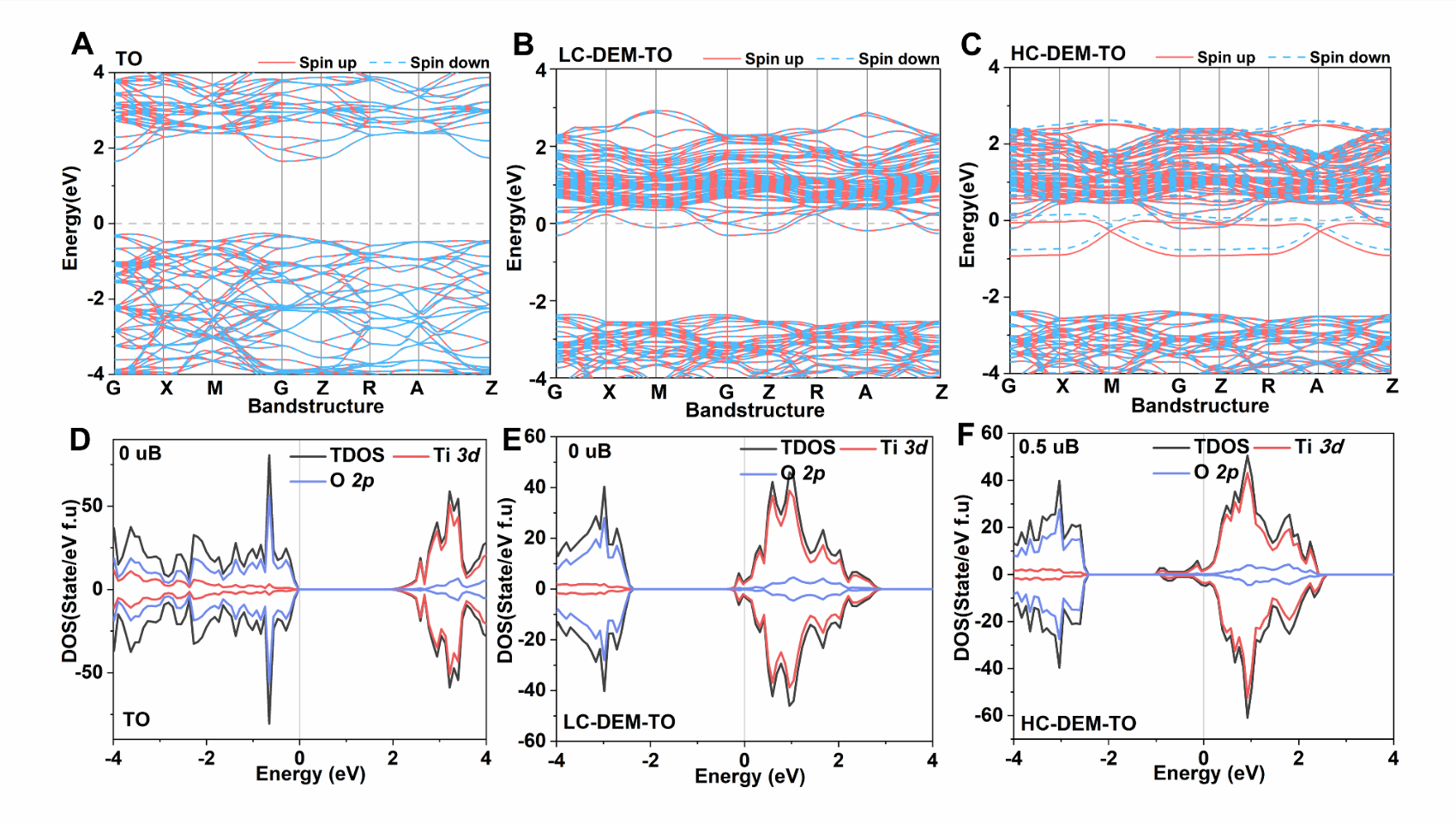


**Figure S6.** The band structure evolution of TO after introducing different oxygen defect concentrations.


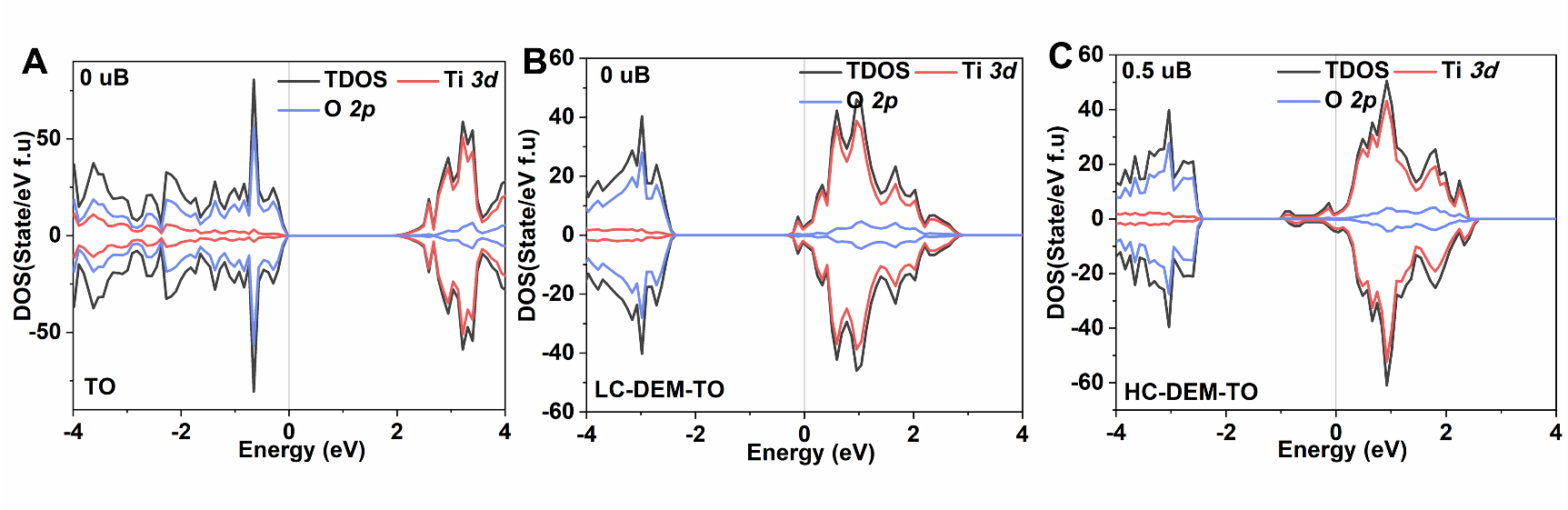


**Figure S7.** The density of states of Ti *3d* and O *2p* in TOs without/with different concentration of oxygen defects.

As illustrated in the **Figure S6-S7**, the magnetic moment and defect state in different oxygen defect concentration were further investigated. In the low concentration of oxygen defects (Here is TiO_1.9375_), no obvious magnetic moment and structural distortion were observed in the band structure, indicating there is no the reduction of Ti^4+^ to Ti^3+^. Further increasing the defect concentrations, admittedly, it can be seen from the band structure that there are detailed defect states and small magnetic moments, indicating the reduction of Ti^4+^ will be partially involved in the systems. However, roughly estimated, each reduction of Ti^4+^ to Ti^3+^ only consumes one electron from the defect but the left one electron will contribute to the delocalized environment (*J. Phys. Chem. C 2011, 115, 7562–7572*). That means most electrons in the low oxygen defect concentration are delocalized instead of the reduction from Ti^4+^ to Ti^3+^.


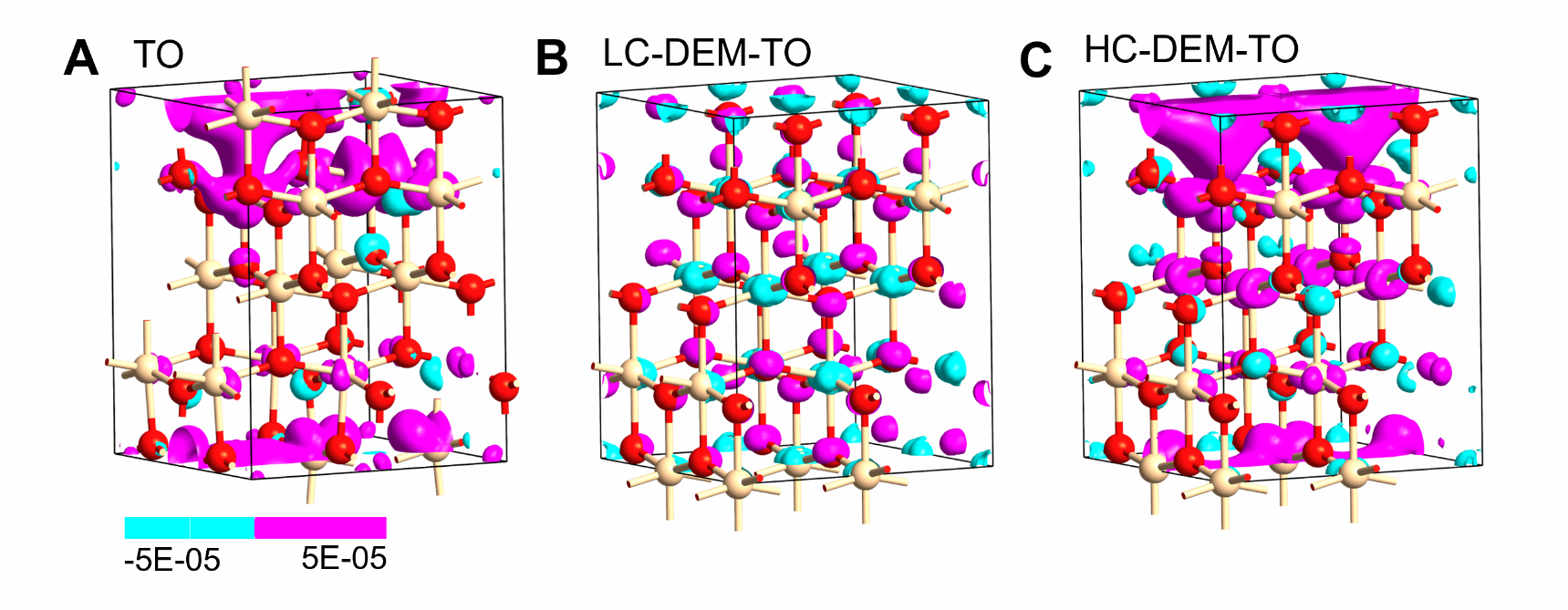


**Figure S8.** 3D electron density distribution on the TO with different concentration of oxygen defects.

The modification of total density of states‌ (TDOS) and ELF for better understanding and observation in displayed in **Figure S8**. In comparison with the pristine TiO_2_, the presence of delocalized electron is obvious as the defect concentration increased.


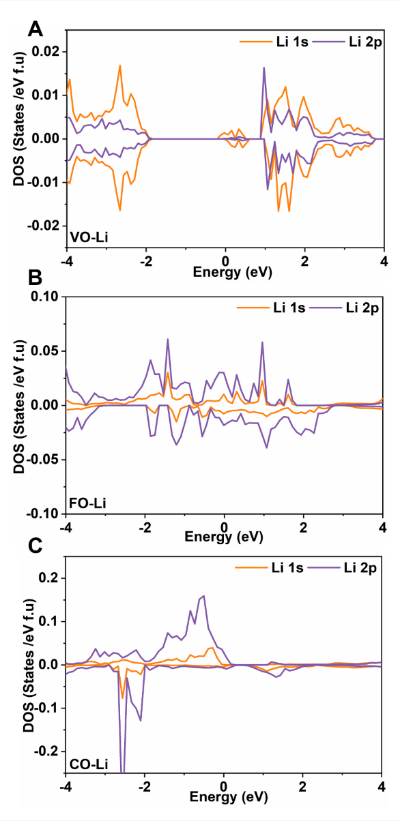


**Figure S9.** The DOS of Li 1s and Li *2p* from the surface atoms in the absorbed configurations of Li species interacted *d*-orbital metal oxides (M=V, Fe, Co).


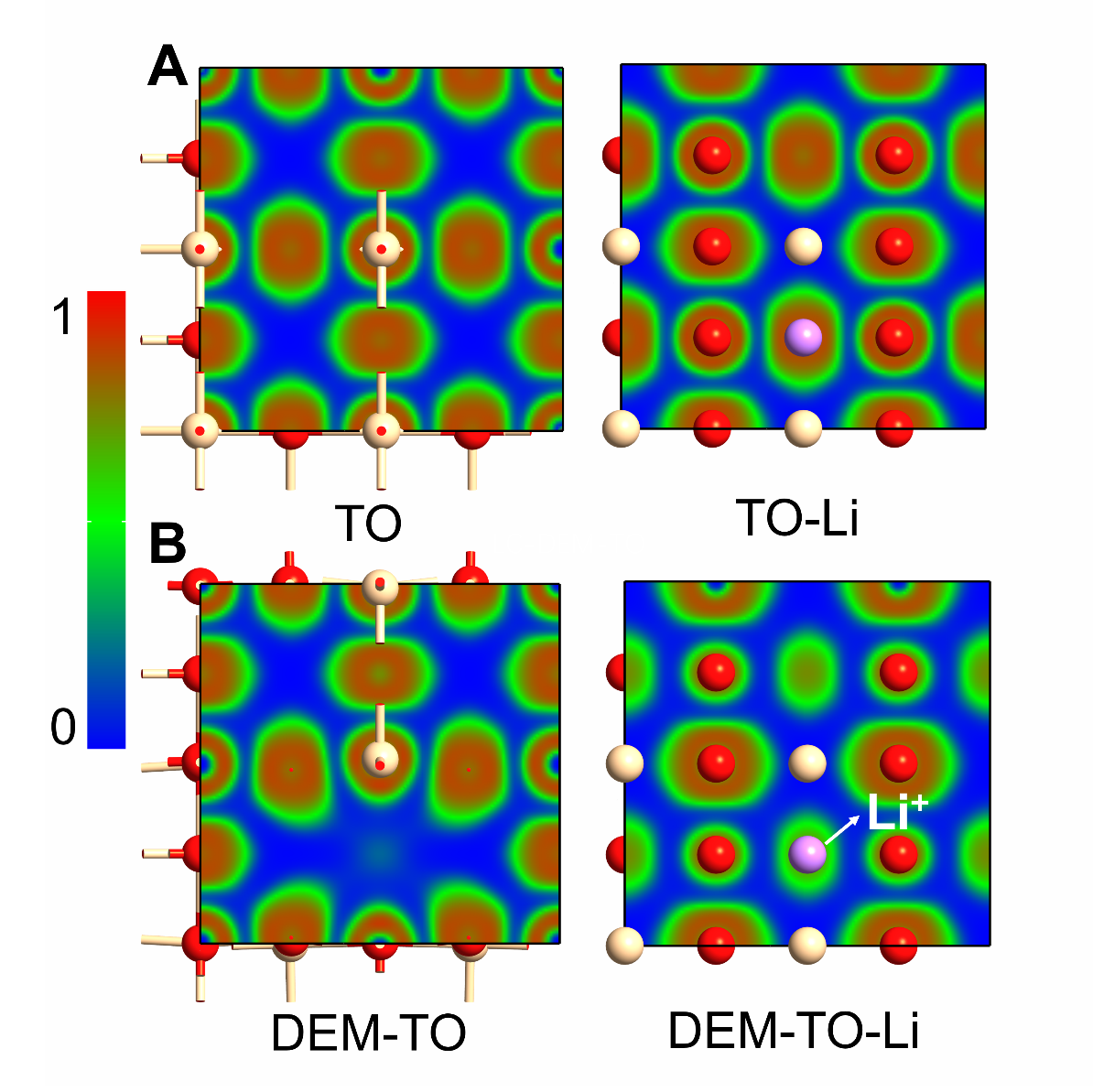


**Figure S10.** 2D slice evolution of ELF before and after interacting with Li.


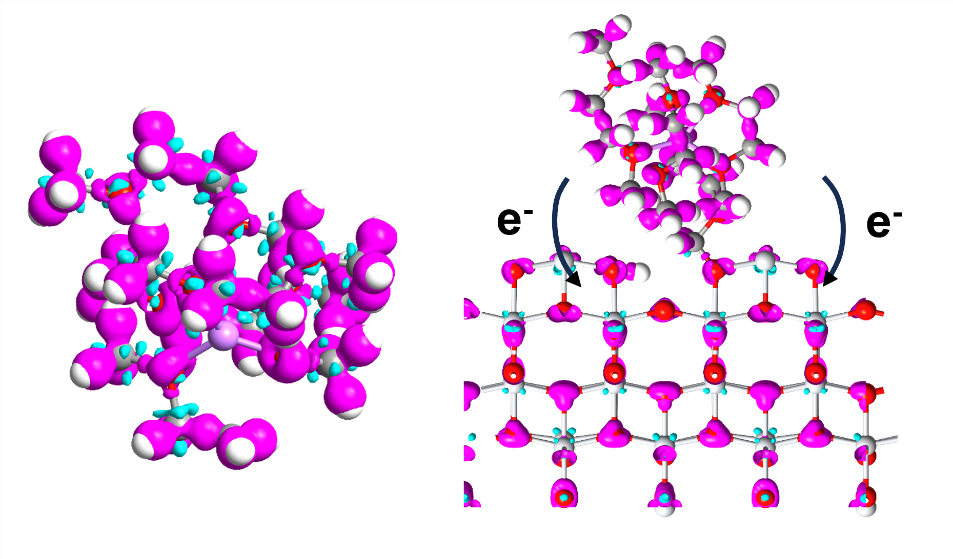


**Figure S11.** The 3D differential electron density transfer from Li(DME)_4_^+^ to DEM-TO@C substrate.


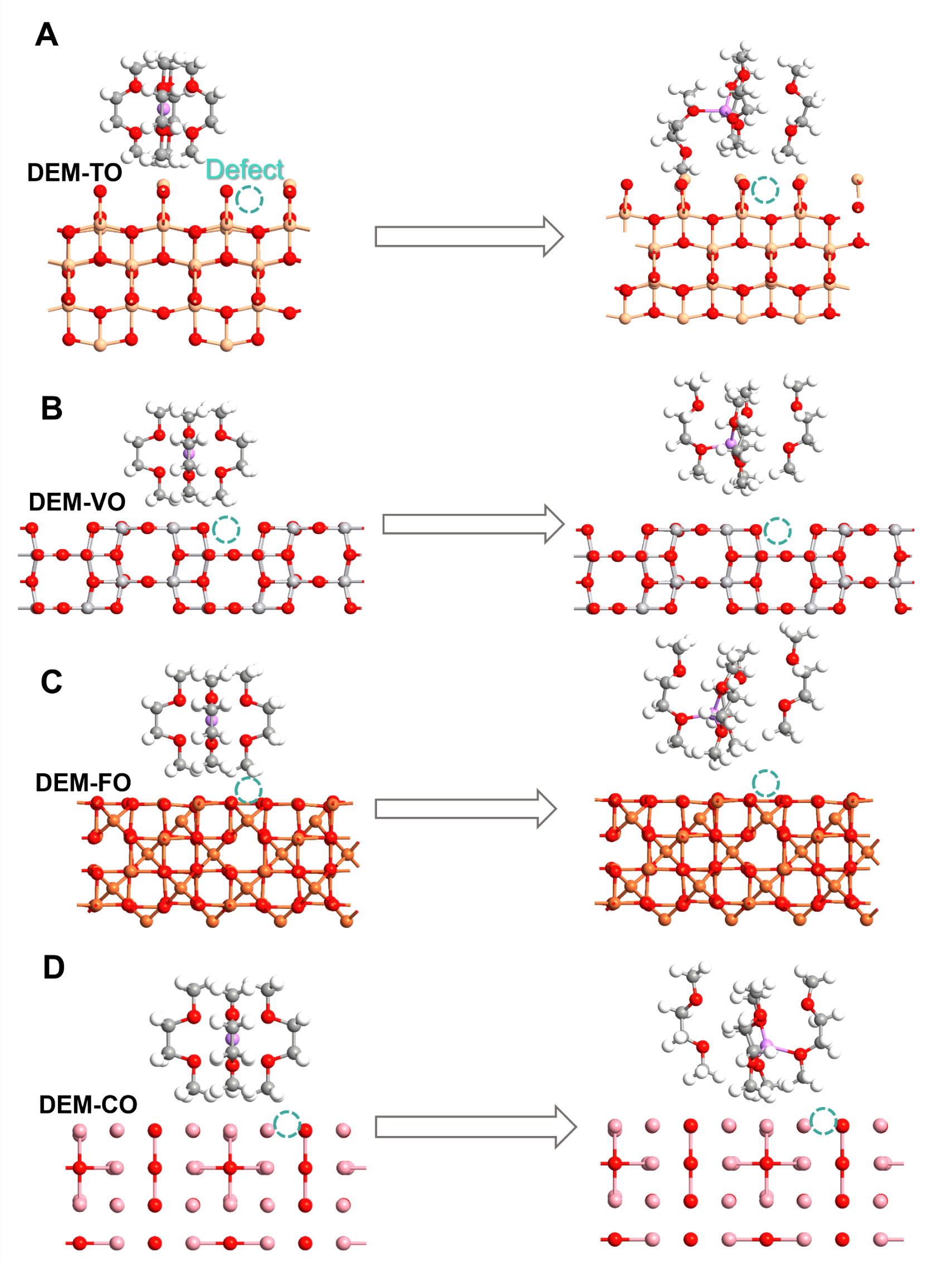


**Figure S12.** Schematic illustration of dissociating Li(DME)_4_^+^ into Li^+^ and DME on delocalization-electron reconfiguration of (A) DEM-TO@C, (B) DEM-VO@C, (C) DEM-FO@C, and (D) DEM-CO@C, respectively.


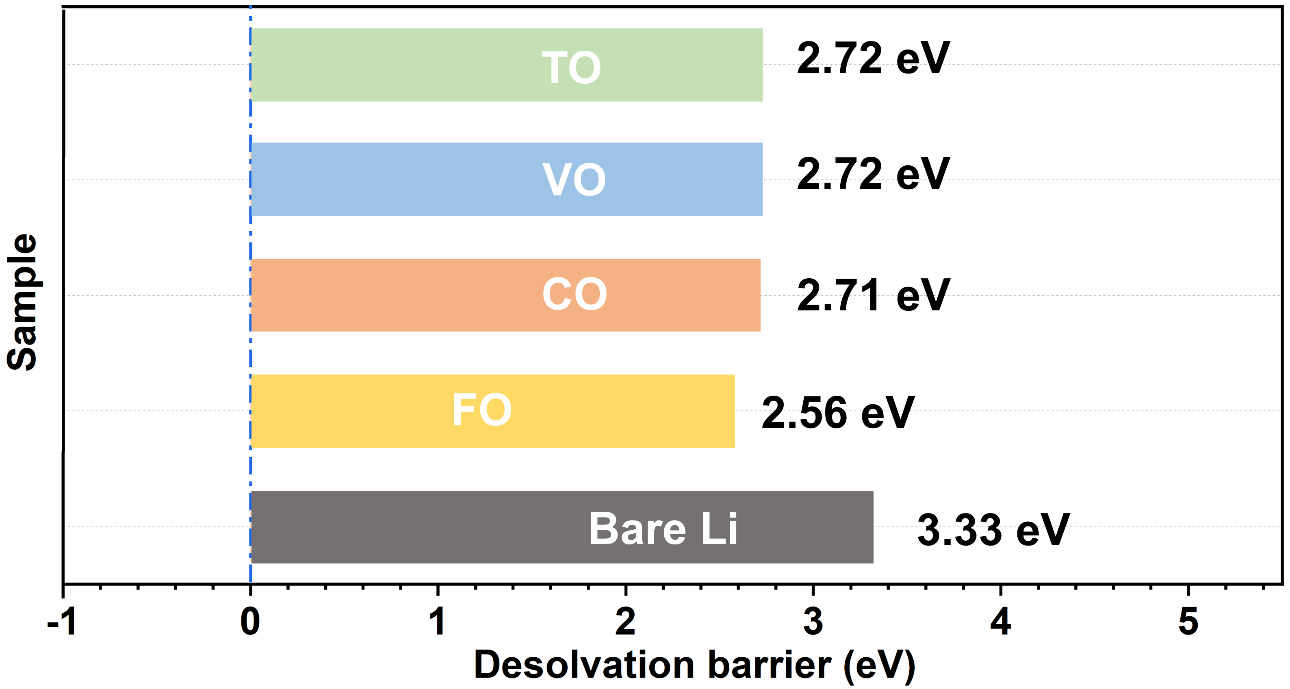


**Figure S13.** Comparison of the desolvation energy barriers of Li(DME)_4_^+^ into Li^+^ and DME on bare Li and different TMOs catalyzers.


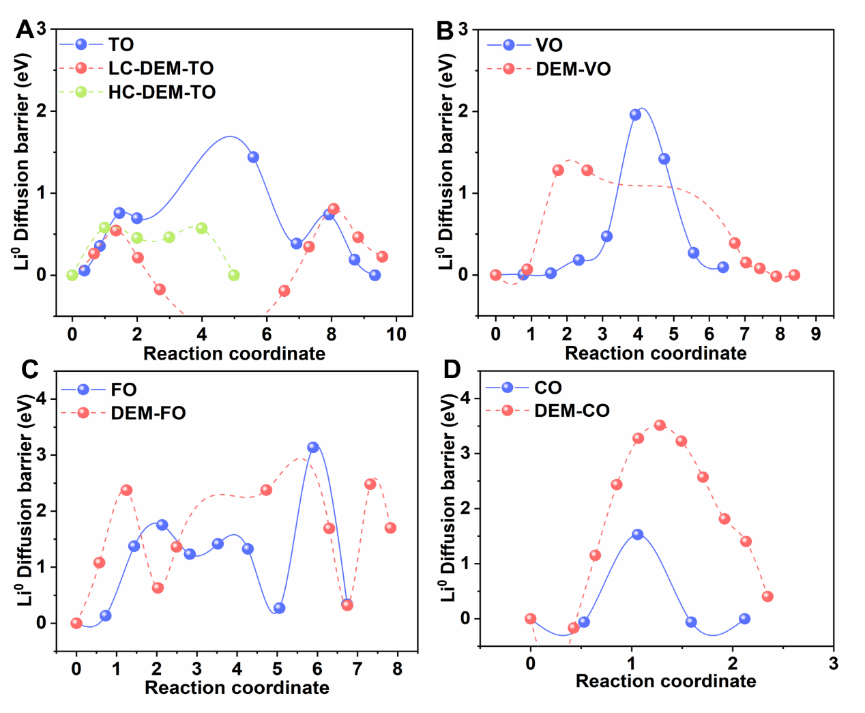


**Figure S14.** Diffusion energy barrier of Li atoms on DEM-TMOs.


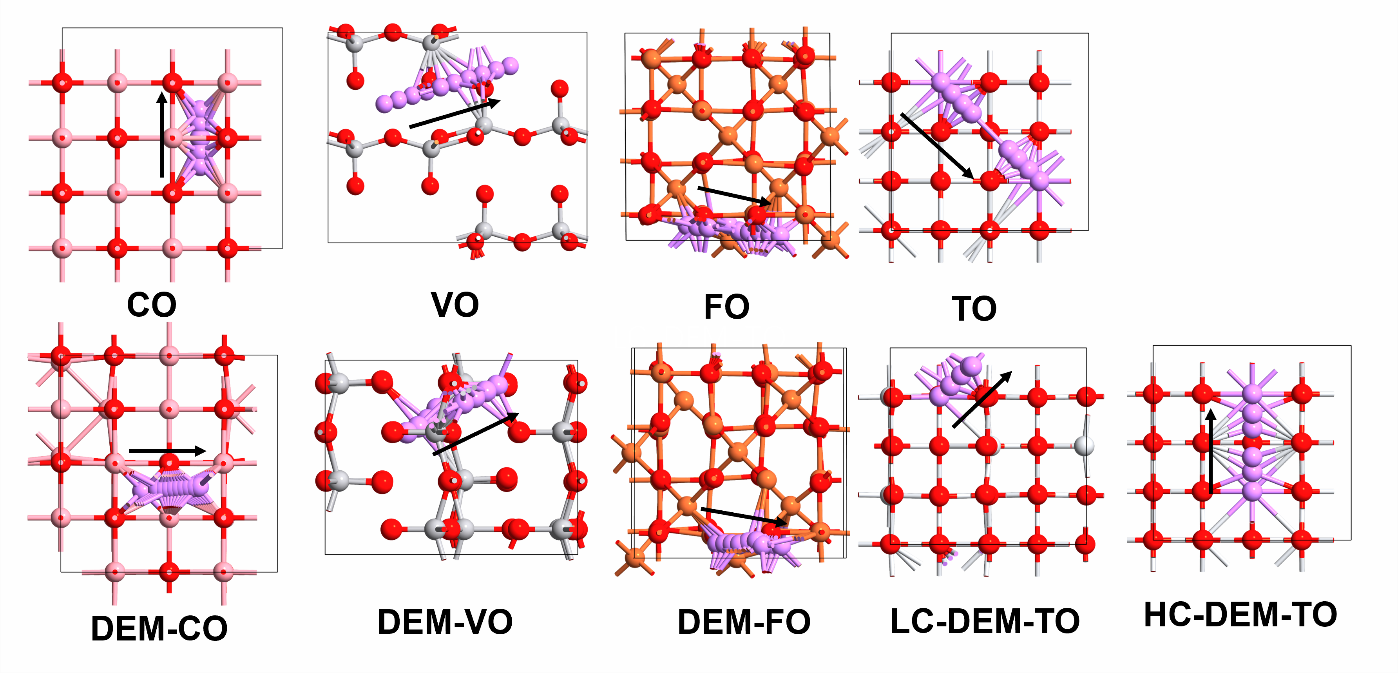


**Figure S15** The transport pathways of Li on TMOs and DEM-TMOs.





**Figure S16.** Comparison of diffusion energy barriers for Li atoms on the different delocalized electron reconstructed catalyzers.


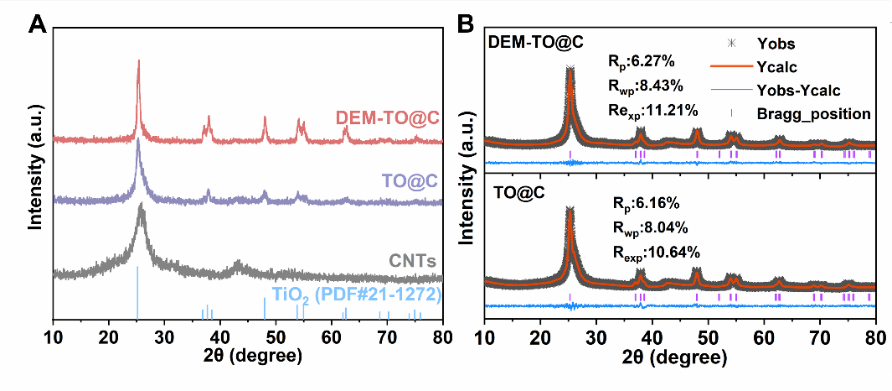


**Figure S17.** (A) XRD pattern and (B) the corresponding Rietveld refinement pattern of the DEM-TO@C and TO@C.


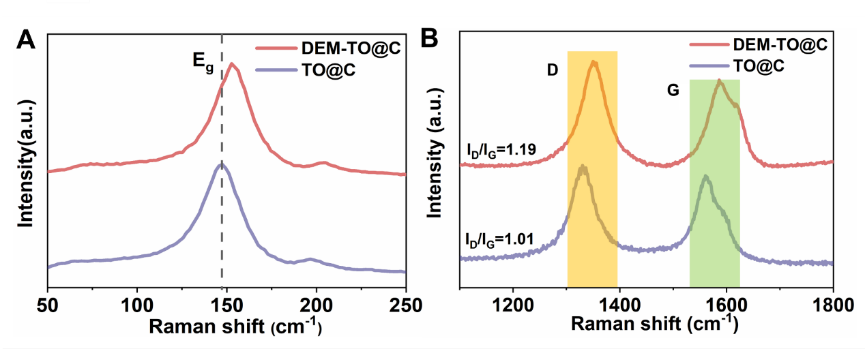


**Figure S18.** Raman comparisons of scattering peak of (A) E_g_ band and (B) D and G band in the DEM-TO@C and TO@C.

As exhibited in **Figure S18**, the I_D_/I_G_ is an index to describe the relative defect amount in the carbon materials, which is corresponding to intensity ratio of feature carbon peaks at D and G band. The increase of I_D_/I_G_ ratio from 1.01 to 1.19 signifies the presence of oxygen-defect induced electron delocalization in the system after hydrogen treatment.


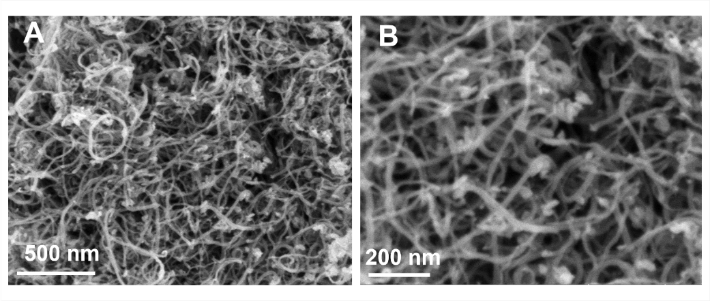


**Figure S19.** SEM images of (A, B) DEM-TO@C nanocomposite.


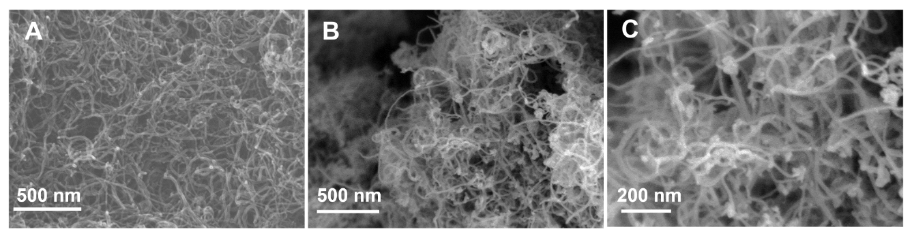


**Figure S20.** SEM images of (A) CNTs, and (B, C) TO@C nanocomposite.


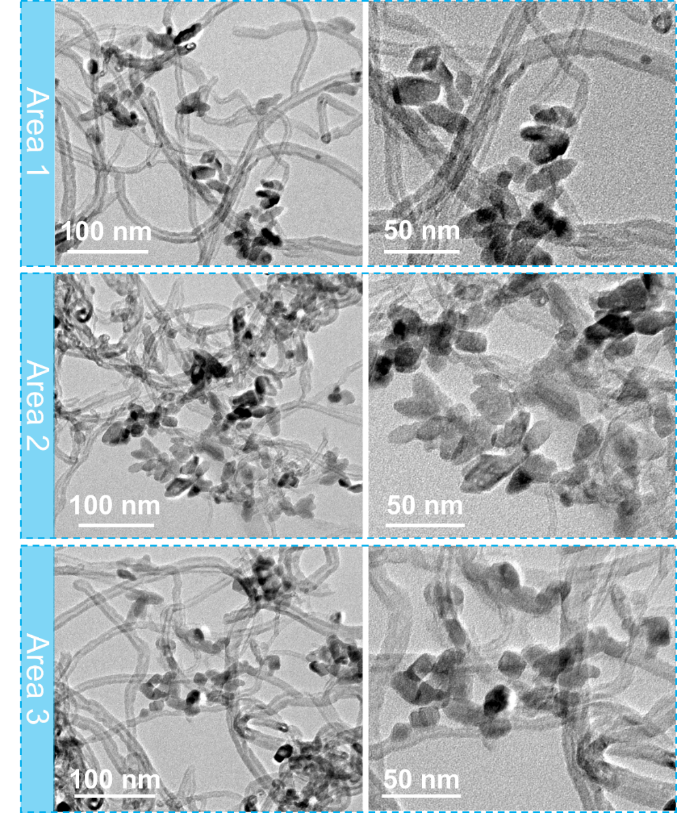


**Figure S21.** TEM images of DEM-TO@C nanocomposite.


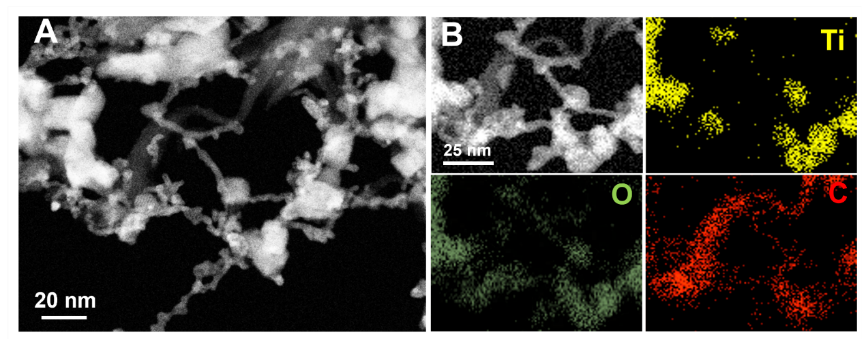


**Figure S22.** (A) STEM image and (B) EDX elemental maps of DEM-TO@C nanocomposite.


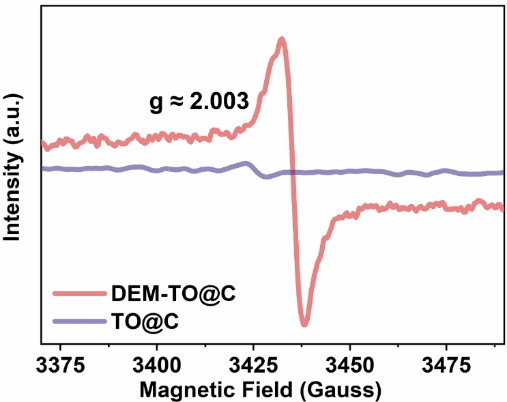


**Figure S23.** EPR spectra for the as-synthesized DEM-TO@C and TO@C nanocomposites.


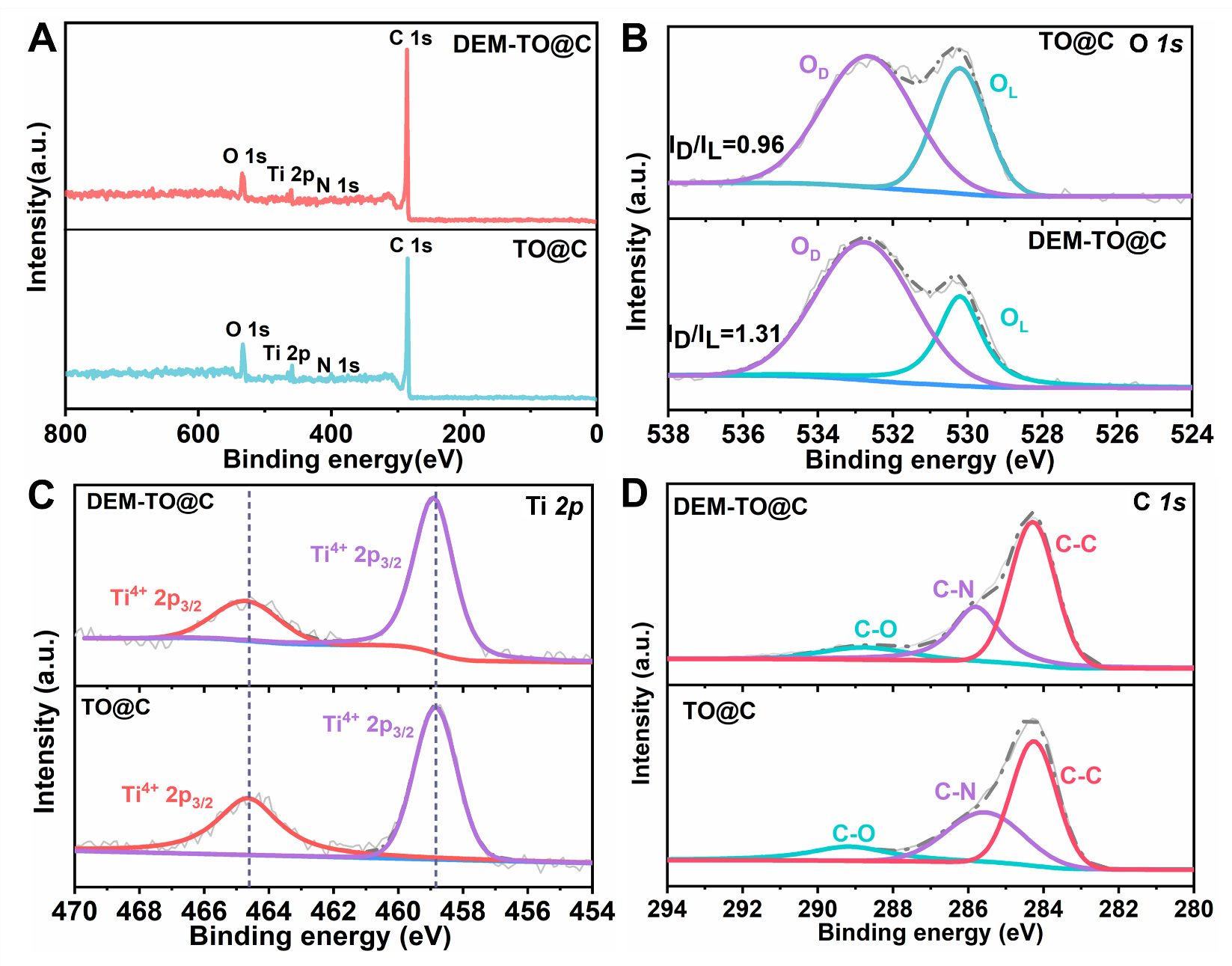


**Figure S24.** Comparison of the (A) XPS full spectra, high-resolution XPS spectra of (B) O 1s, (C) Ti 2p and (D) C 1s between the DEM-TO@C and TO@C.





**Figure S25.** Mott-Schottky plots carried on defective DEM-TO@C and TO@C symmetric cells at 1 kHz frequency using 1 mol L^-1^ Na_2_SO_4_ electrolyte.


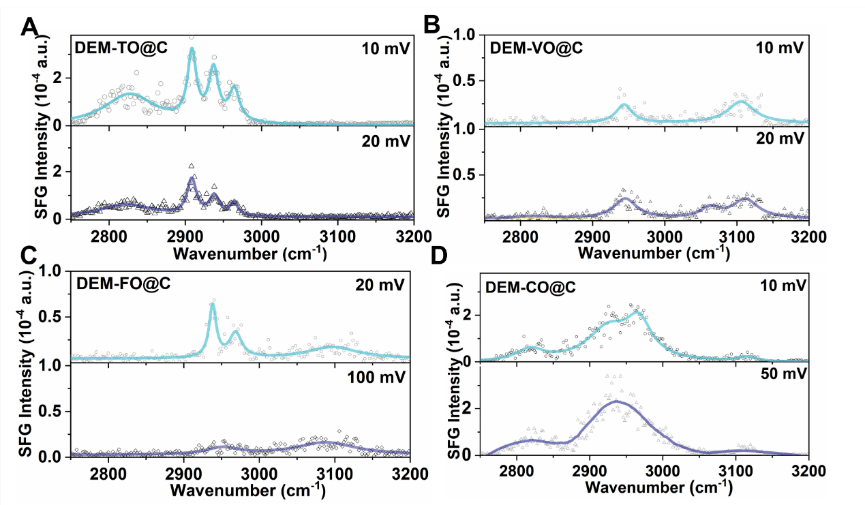


**Figure S26.** The SFG spectra of solvated Li^+^ structure at the C-H region with/without DEM-TMOs@C catalyzers under different bias voltage on.





**Figure S27.** The variation trend of SFG intensity under catalysis of DEM-TMOs with the increase of bias voltage.





**Figure S28.** The calculation method of SFG intensity difference of the main C-H peak with/without bias voltage.

The compositional variation of DEM-TMOs catalyzers induces systematic shifts in C-H vibrational peaks, owing to electronic modulation of ordination interfacial charge transfer between catalytic centers and adsorbed solvent molecules. Therefore, the C-H vibrational peaks are quantitatively compared based on the dominant stretching mode with strongest main peak located at ~2970 cm^-1^. In this way, the SFG peak intensity difference was quantified before and after applying bias voltage into the in-situ systems, as shown in **Figure S28**.


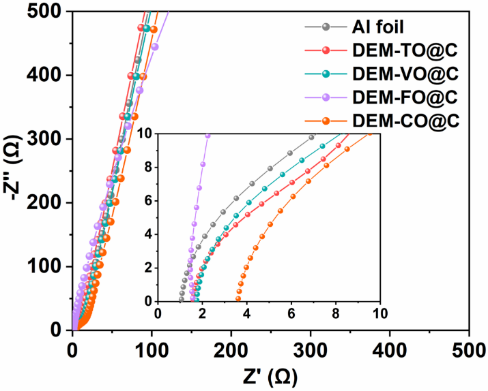


**Figure S29.** Ionic conductivity test on the DEM-TMOs@C.

The ion conductivity (σ) was calculated as the following equation:

σ =𝐿/𝑅_𝑏_𝑆

where L represents the thickness of the DEM-TMOs@C layer, R_b_ is the resistance of the layer tested by EIS and S is the effective contacting area.


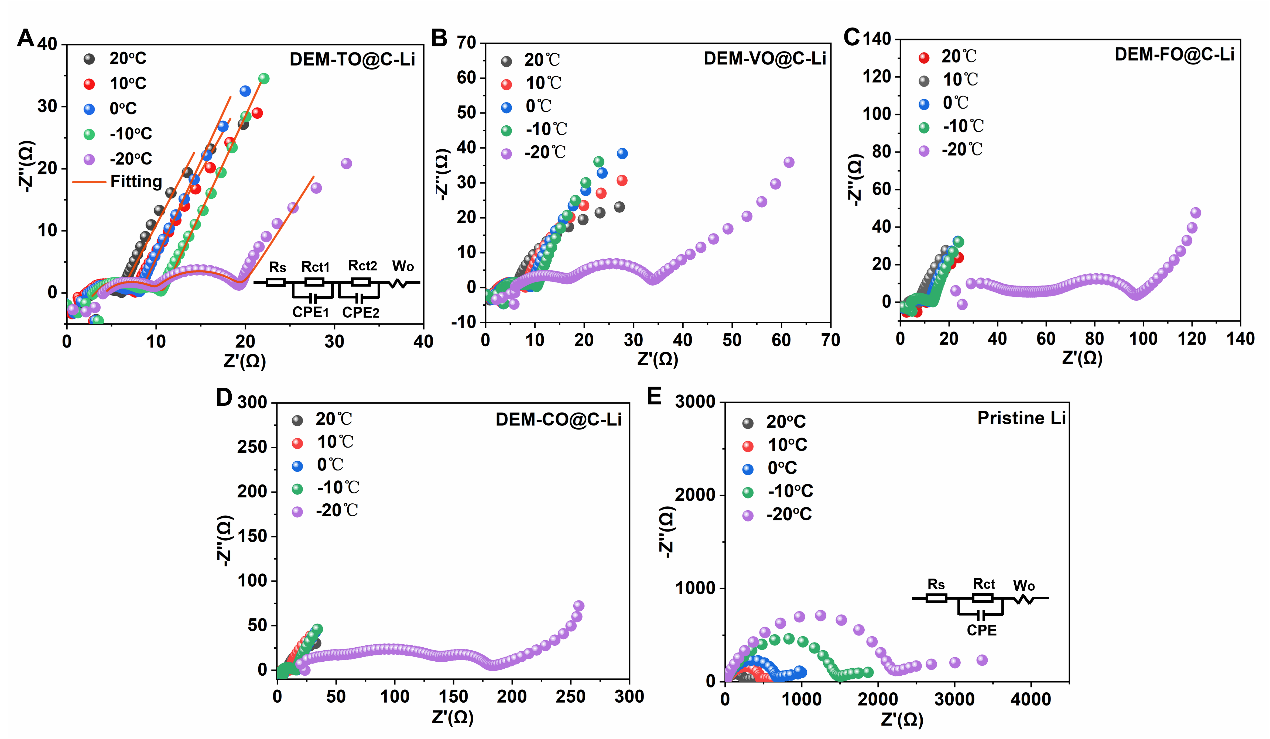


**Figure S30.** Comparison of EIS for Li||Li symmetric cells with/without DEM-TMOs@C under shifting temperature: (A) DEM-TO@C-Li; (B) DEM-VO@C-Li; (C) DEM-FO@C-Li; (D) DEM-CO@C-Li and (E) Pristine Li.


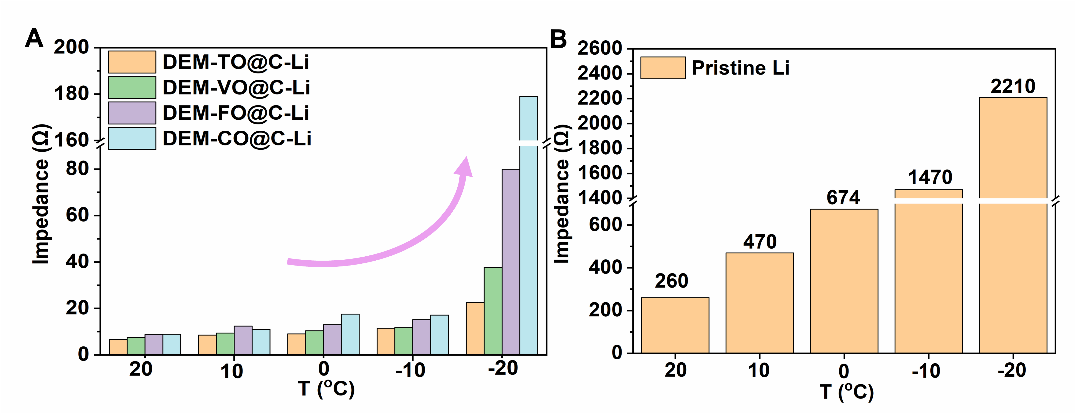


**Figure S31.** Comparing charge interfacial impedances of Li||Li symmetric cells with DEM-TMOs@C-Li and (B) Pristine Li under shifting temperature.


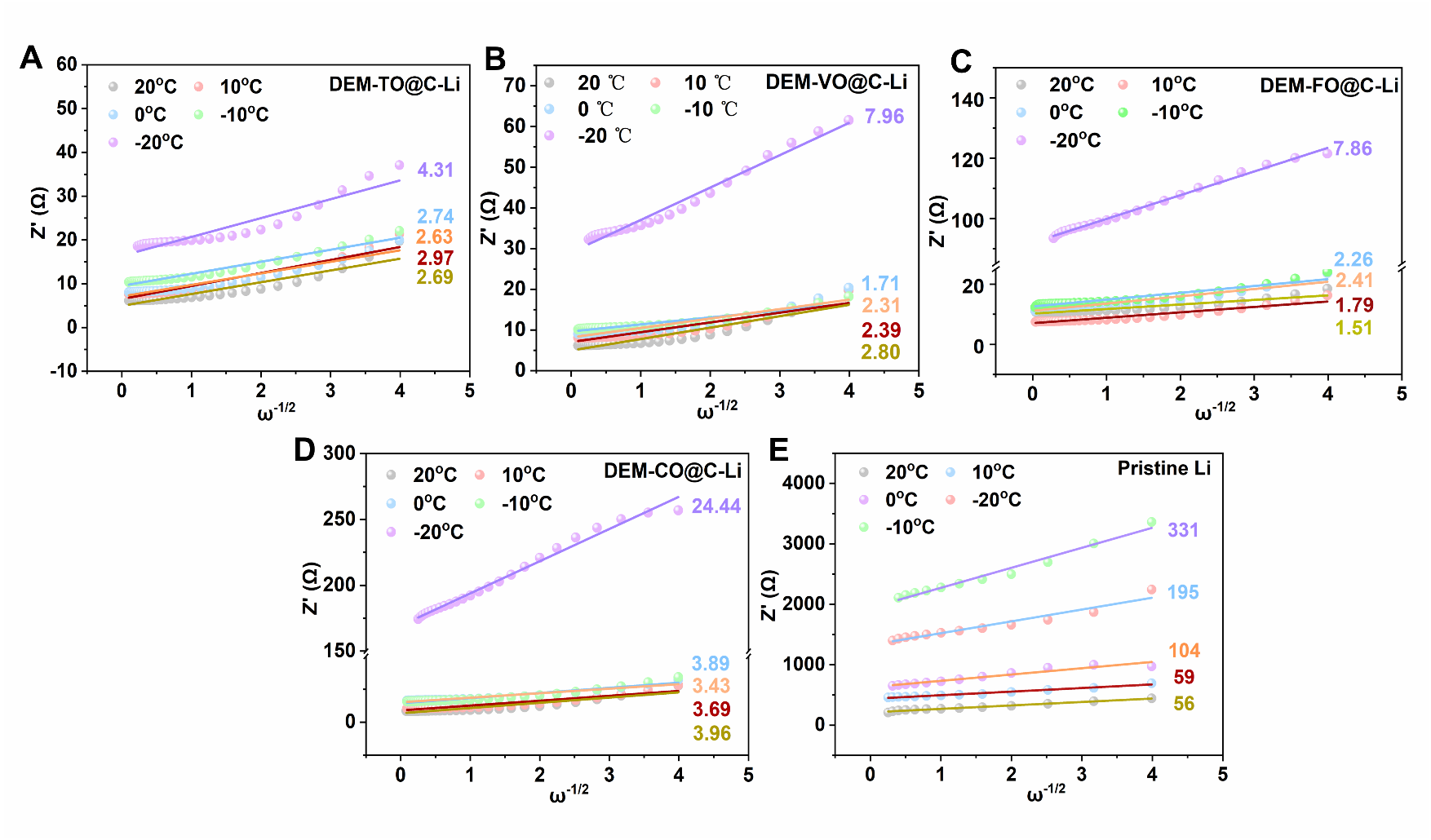


**Figure S32.** Comparison of Li^+^ diffusion kinetics for Li||Li symmetric cells with/without DEM-TMOs@C under shifting temperature.


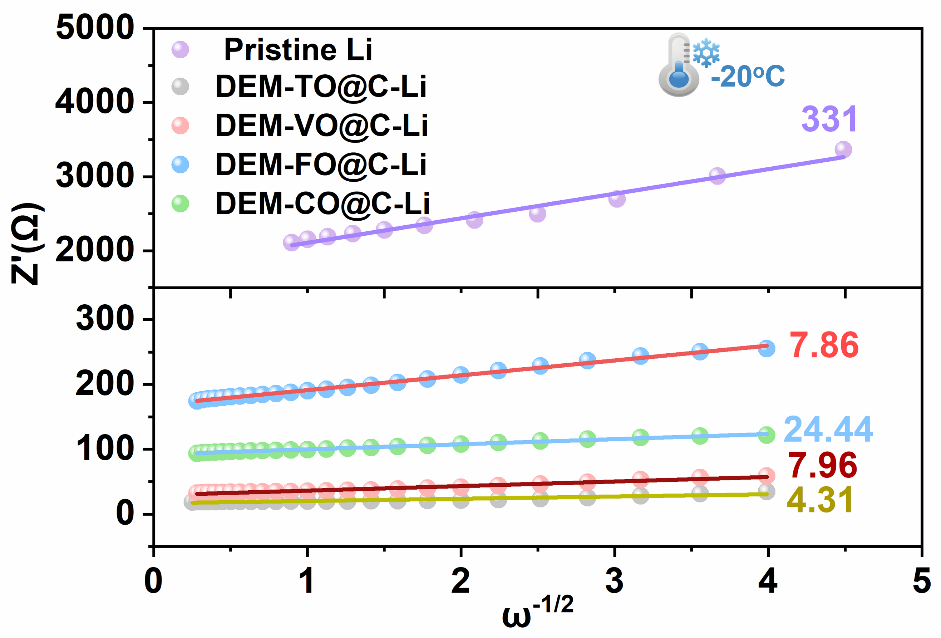


**Figure S33.** Comparison of Li-ion transport kinetics with/without DEM-TMOs@C under low temperature of -20^o^C.


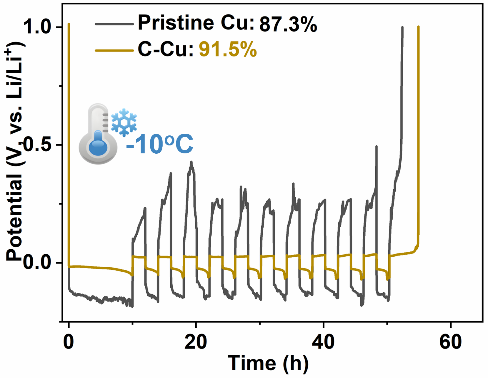


**Figure S34.** Aurbach CE test on CNT-Cu (denoted as C-Cu) and pristine Cu asymmetrical cell.


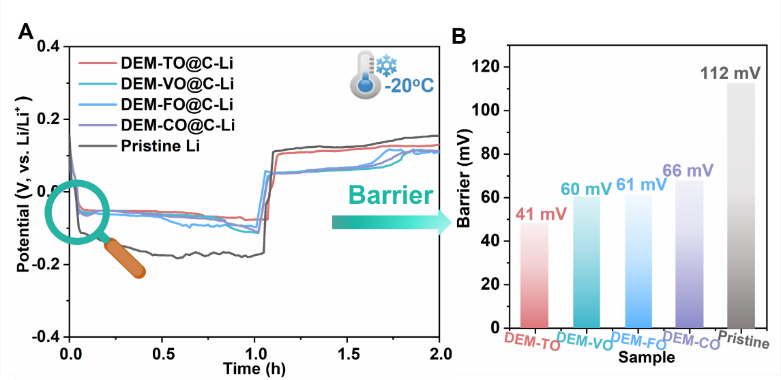


**Figure S35.** Comparison of initial Li nucleation overpotentials on the DEM-TMOs@C-Li electrodes at 1 mA cm^-2^ with 1 mA h cm^-2^.


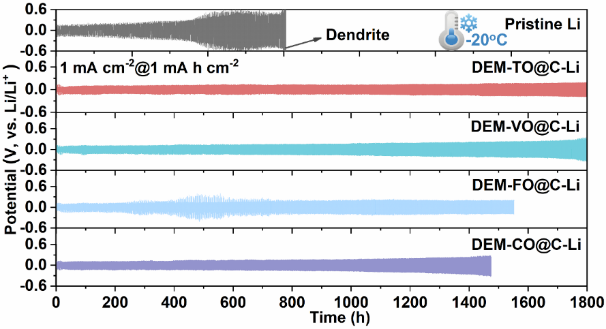


**Figure S36.** Constant galvanostatic plating/stripping stability under -20°C at 1 mA cm ^-2^ with 1 mA h cm ^-2^.


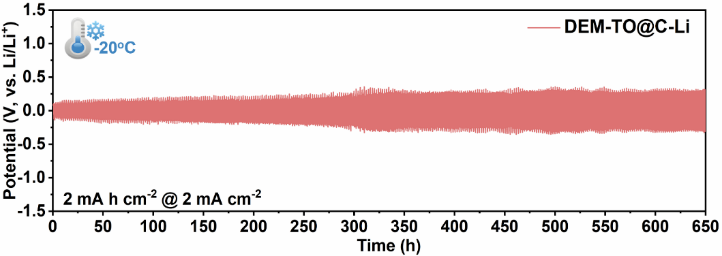


**Figure S37.** Galvanostatic plating/stripping stability of the DEM-TO@C-Li electrode under -20°C at 2 mA cm^-2^ with 2 mA h cm^-2^.


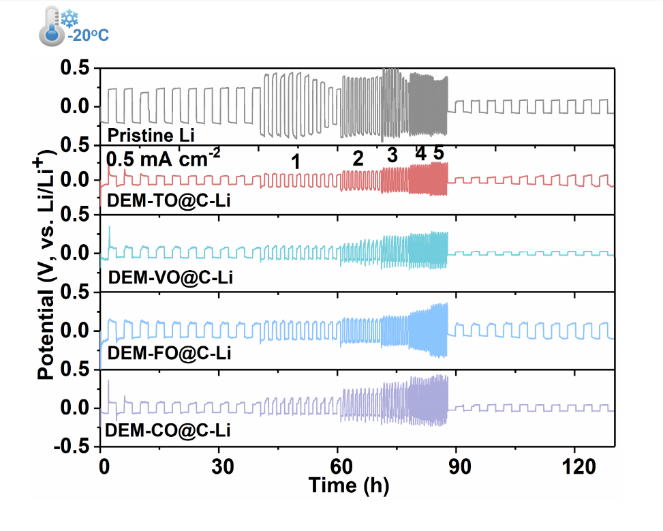


**Figure S38.** Comparison of rate performance of DEM-TMOs@C-Li electrodes at shifting current densities.


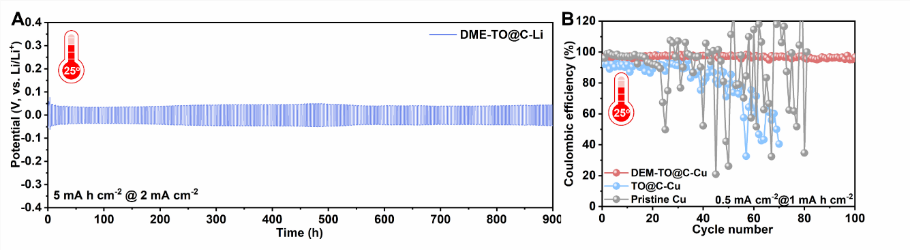


**Figure S39.** (A) Galvanostatic plating/stripping stability based on the DEM-TO@C-Li electrode at 2 mA cm^-2^ with 5 mA h cm^-2^ and (B) Coulombic efficiencies of Li||Cu cells based on the DEM-TO@C-Cu electrode at 0.5 mA cm^-2^ with 1 mA h cm^-2^ under room temperature.


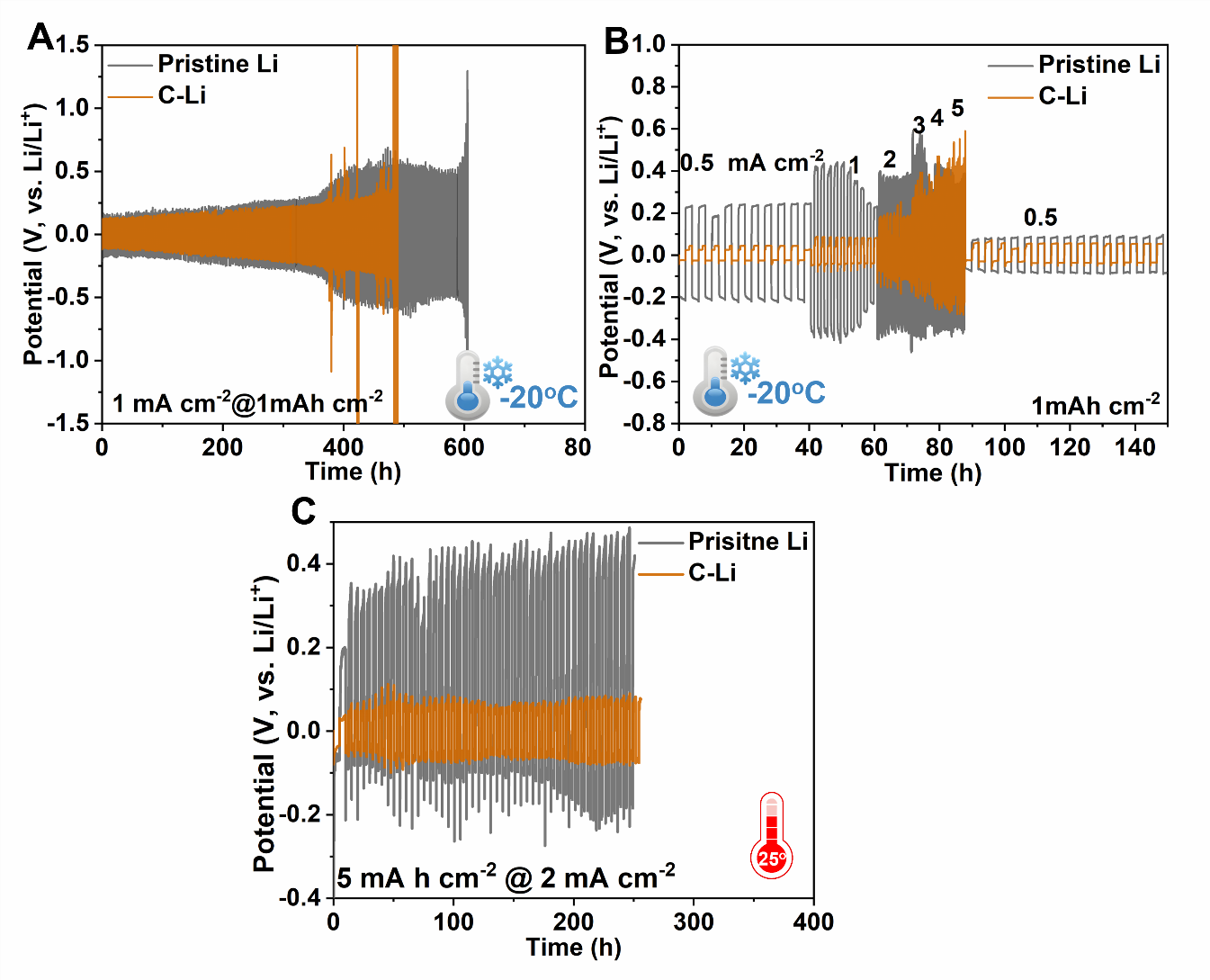


**Figure S40.** Comparison of (A) constant galvanostatic plating/stripping stability at 1 mA cm^-2^ with 1 mA h cm^-2^ and (B) rate performance at shifting current densities of C-Li and pristine Li electrodes under -20°C; (C) Galvanostatic plating/stripping stability based on the C-Li and pristine Li electrodes at 2 mA cm^-2^ with 5 mA h cm^-2^.


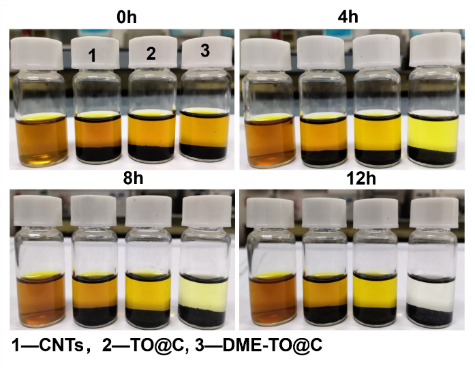


**Figure S41.** Digital photos of the DEM-TO@C and the TO@C nanocomposites soaked in 5 mmol L^-1^ Li_2_S_8_ solution: after sufficient exposure to the adsorbents for (A) 2 h; (B) 4 h; (C) 8 h and (D) 12 h.

**

**

**Figure S42.** Current response at the constant potential of 2.09 V corresponding to a liquid/solid phase conversion threshold on DEM-TO@C and TO@C catalytic surface.





**Figure S43.** Comparison of EIS profiles carried out on different Li-S full batteries.


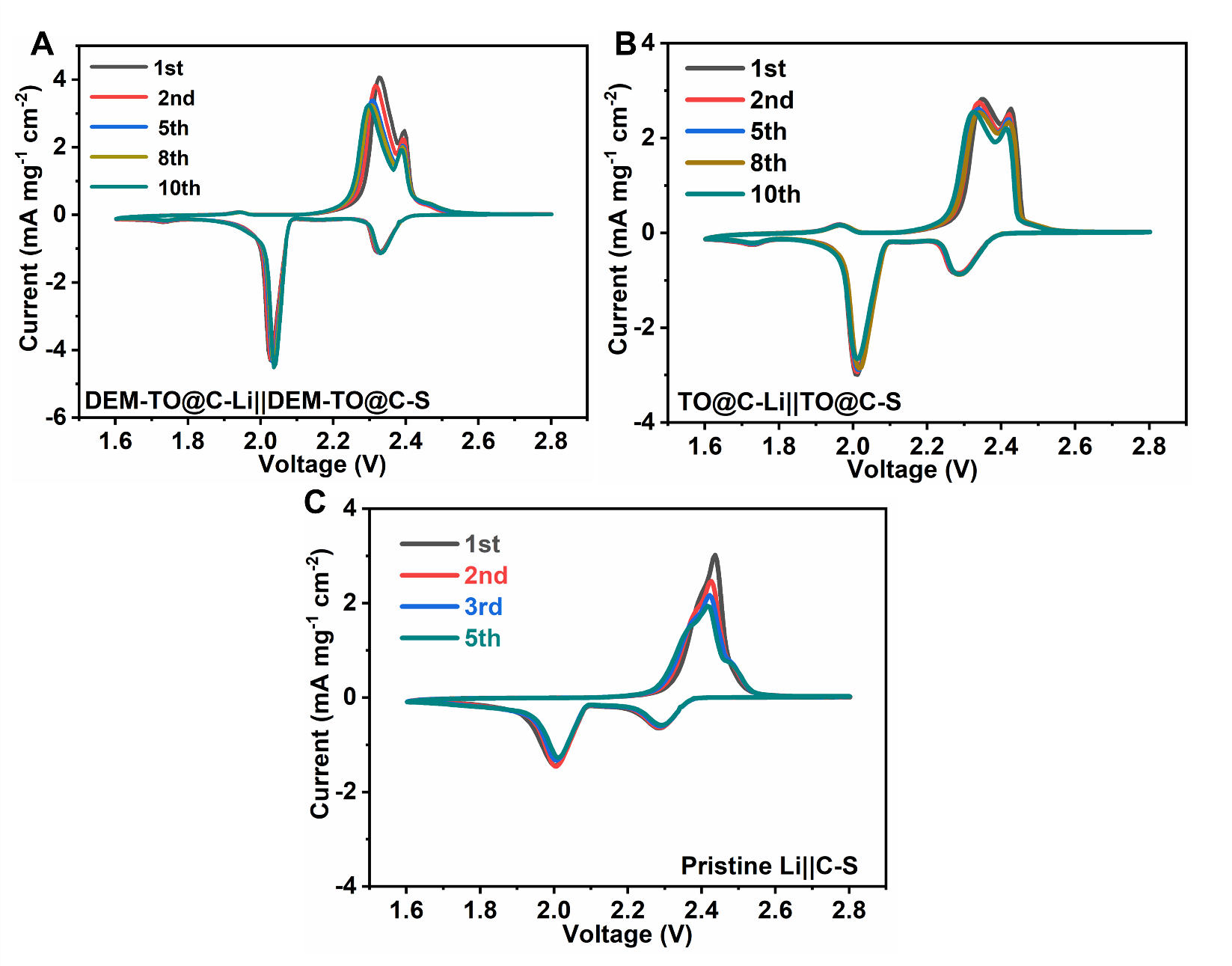


**Figure S44.** Comparison of CV curves on three different Li-S full batteries at 0.1 mV s^-1^ within the scan range from 1.6 to 2.8 V (vs. Li/Li^+^).


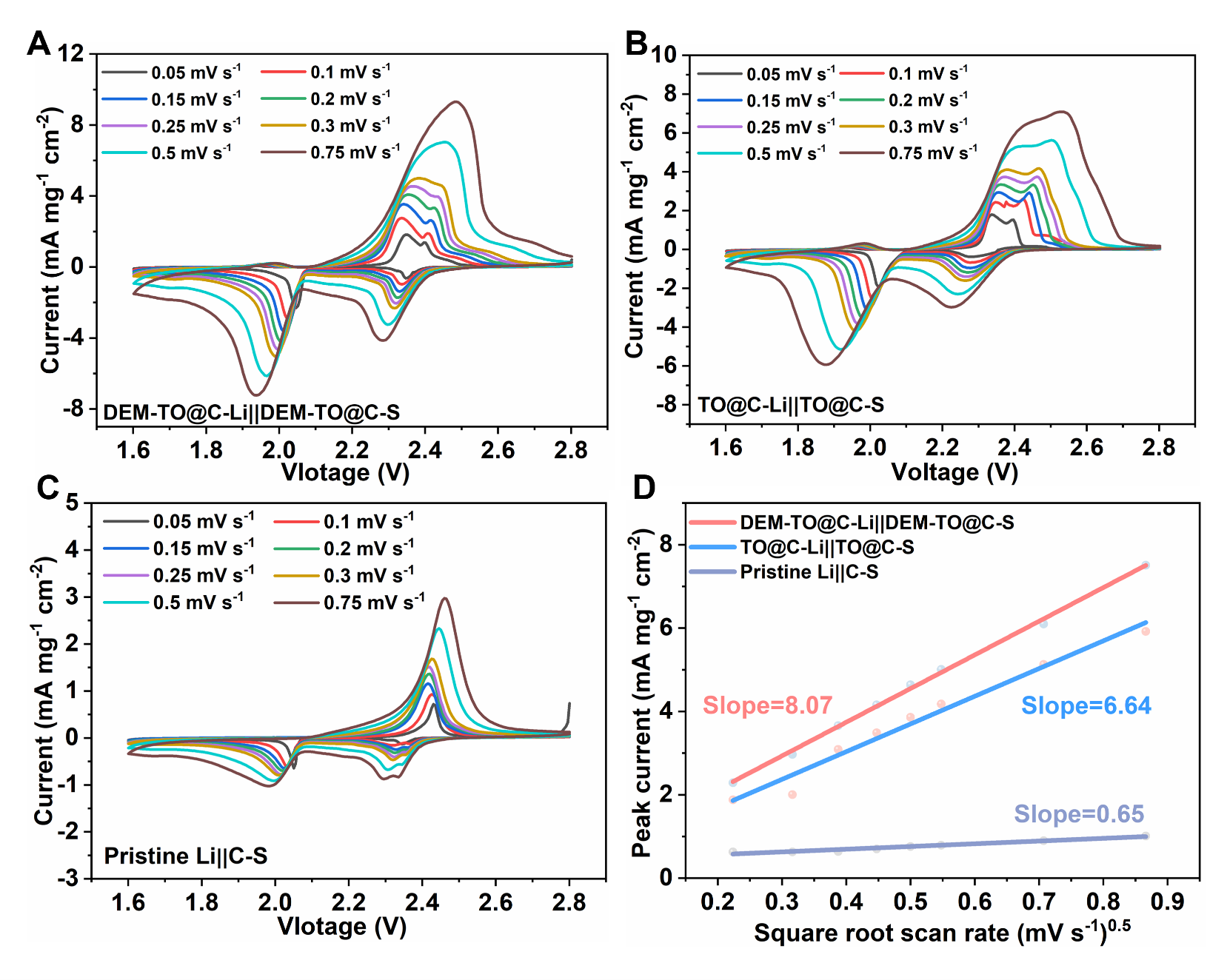


**Figure S45.** Scan rate-dependent CV profiles of the (A) DEM-TO@C-Li||DEM-TO@C-S; (B) TO@C-Li||TO@C-S and (C) Pristine Li||C-S full batteries; (D) Plot of the peak current recorded at the second cathodic current peak (Li_2_S_4_→Li_2_S) versus the square root of the scan rates.


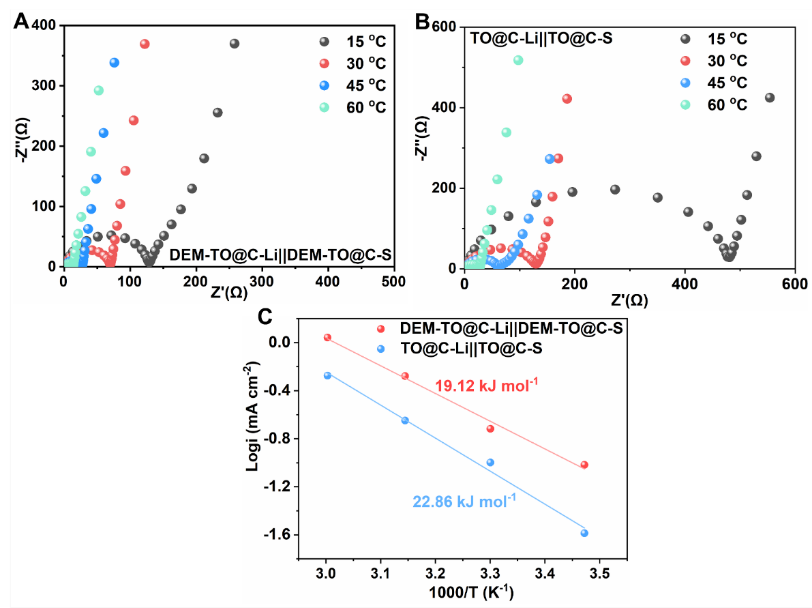


**Figure S46.** EIS profiles carried out on different Li-S full batteries under variable operation temperature: (A) DEM-TO@C-Li||DEM-TO@C-S and (B) TO@C-Li||TO@C-S; (C) Comparison of E_a_ calculated by Arrhenius equation on the different Li-S full batteries.


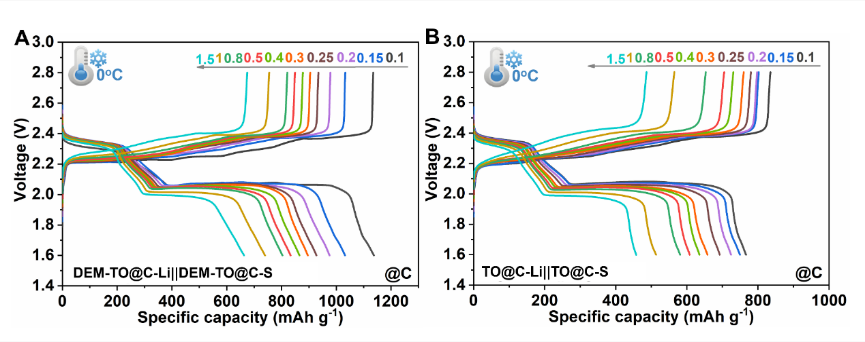


**Figure S47.** Galvanostatic charge and discharge curves for (A) DEM-TO@C-Li||DEM-TO@C-S and (B) TO@C-Li||TO@C-S cells at incremental current rates under 0^o^C.


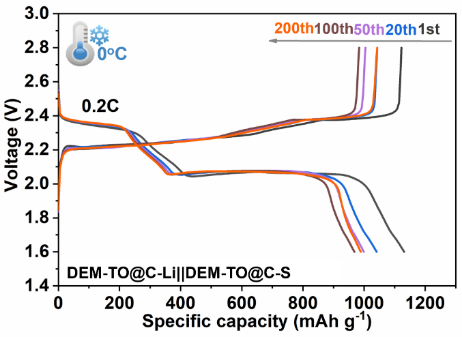


**Figure S48.** Voltage-capacity profiles of the DEM-TO@C catalyzed Li-S full battery within 200 cycles at 0.2 C under 0°C.


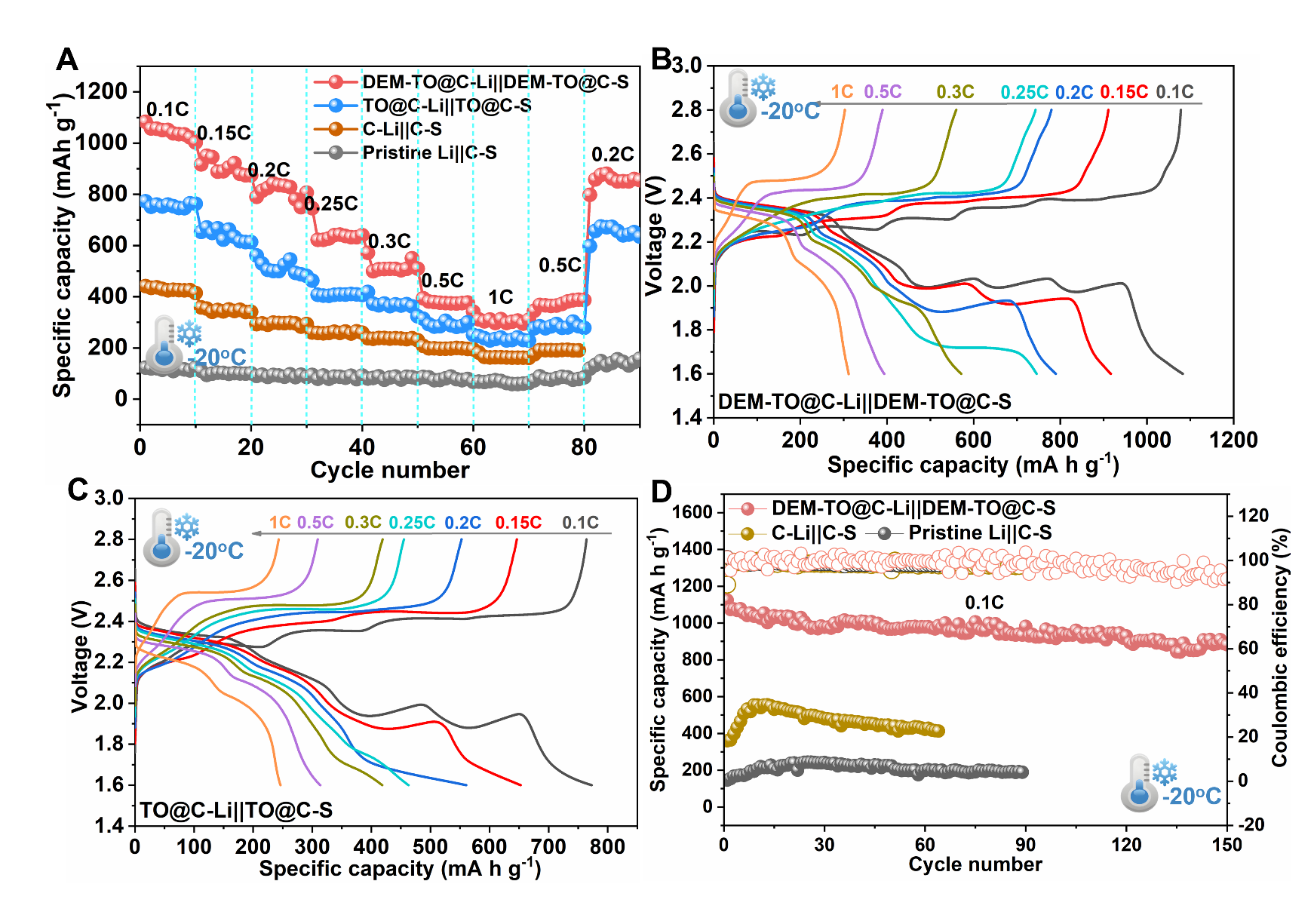


**Figure S49.** (A) Comparisons in rate performance of catalyzed full batteries under -20°C; Galvanostatic charge and discharge curves for (B) DEM-TO@C-Li||DEM-TO@C-S and (C) TO@C-Li||TO@C-S cells at incremental current rates under -20^o^C; (D) Cycling performance of the DEM-TO@C catalyzed full battery at 0.1 C under -20°C.


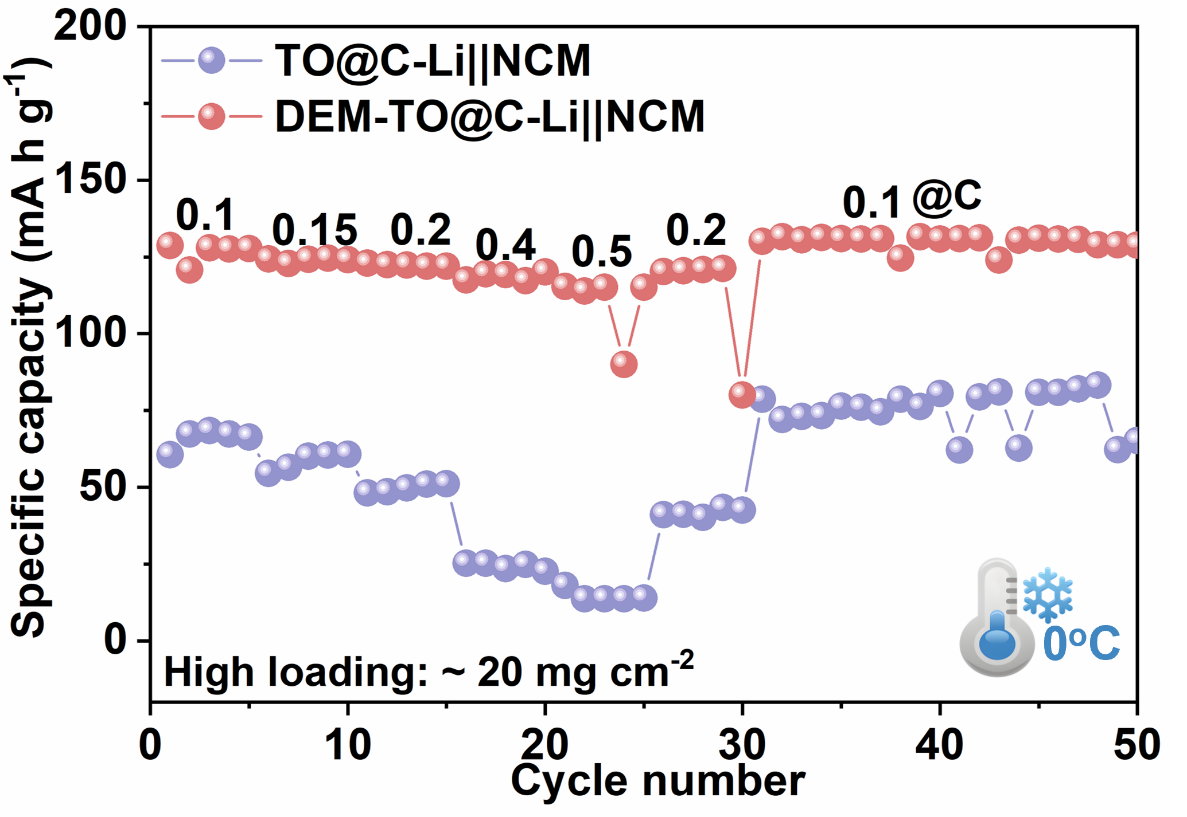


**Figure S50.** Comparison in rate performance between catalyzed DEM-TO@C-Li||NCM and TO@C-Li||NCM full batteries with ultra-high loading of active materials charged to 4.2V under 0°C.


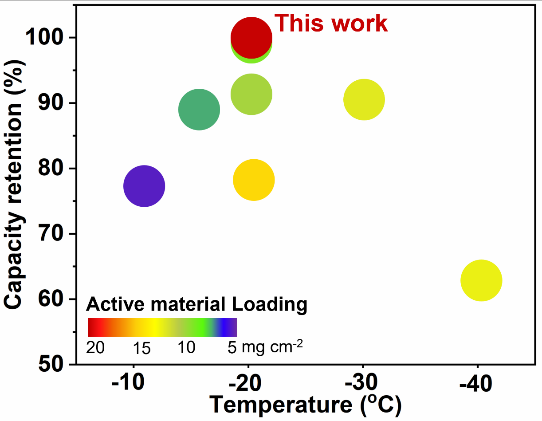


**Figure S51.** Comparison of the capacity retention and active material loading at low temperatures with reported literatures.


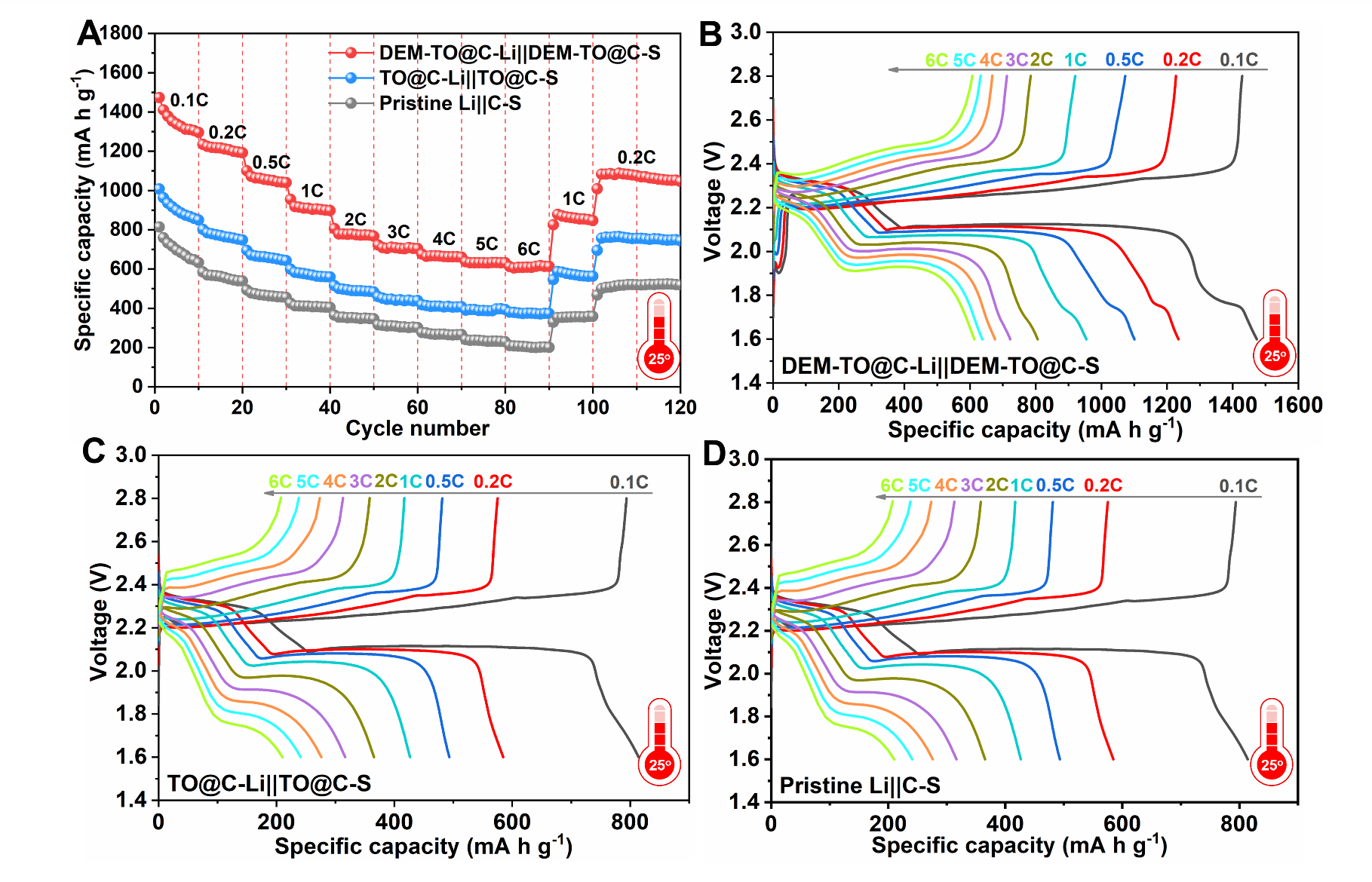


**Figure S52.** (A) Rate performances of different Li-S full cells at room temperature; Galvanostatic charge and discharge curves for (B) DEM-TO@C-Li||DEM-TO@C-S, (C) TO@C-Li||TO@C-S and (D) Pristine Li||C-S cells with incremental current rates at room temperature.


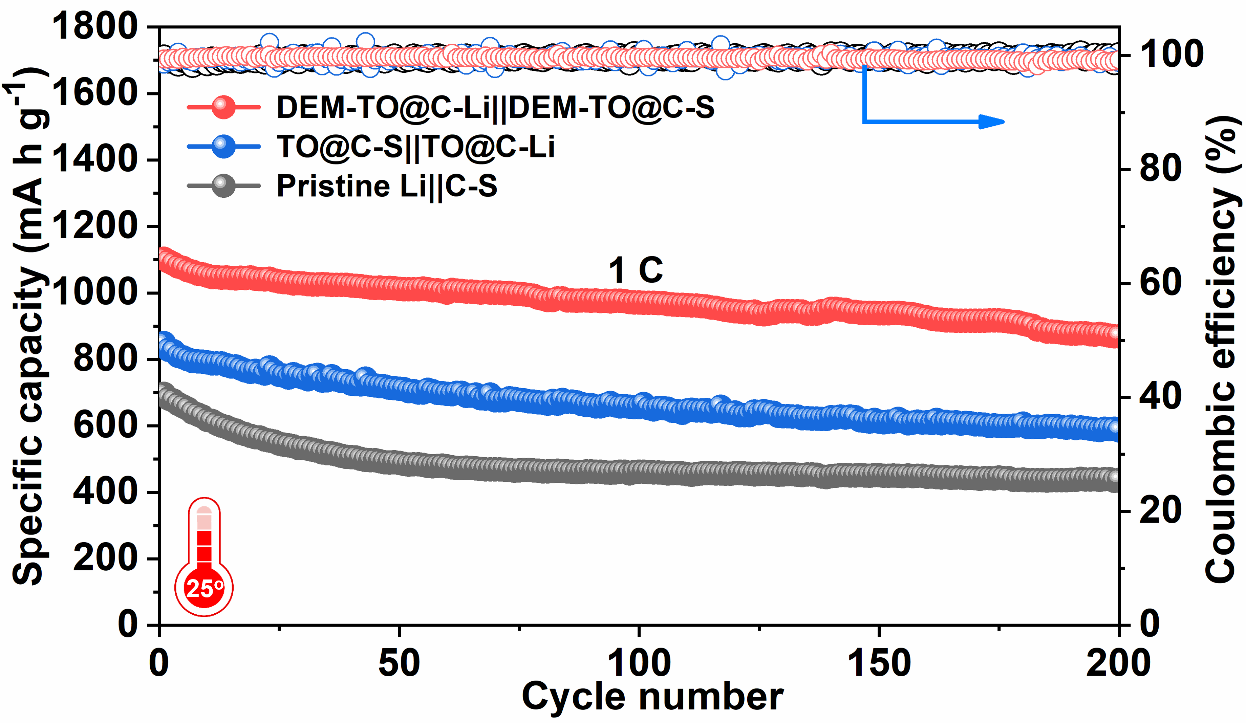


**Figure S53.** Cycling performances of different Li-S full cells at 1C under room temperature.


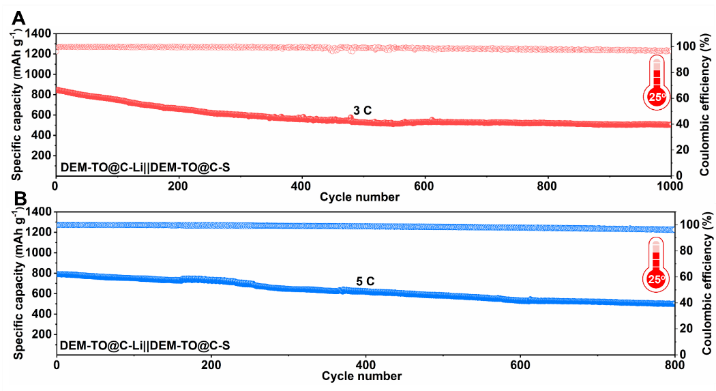


**Figure S54.** Cycling performances of DEM-TO@C-Li||DEM-TO@C-S full cells at (A) 3C and (B) 5C under room temperature, respectively.


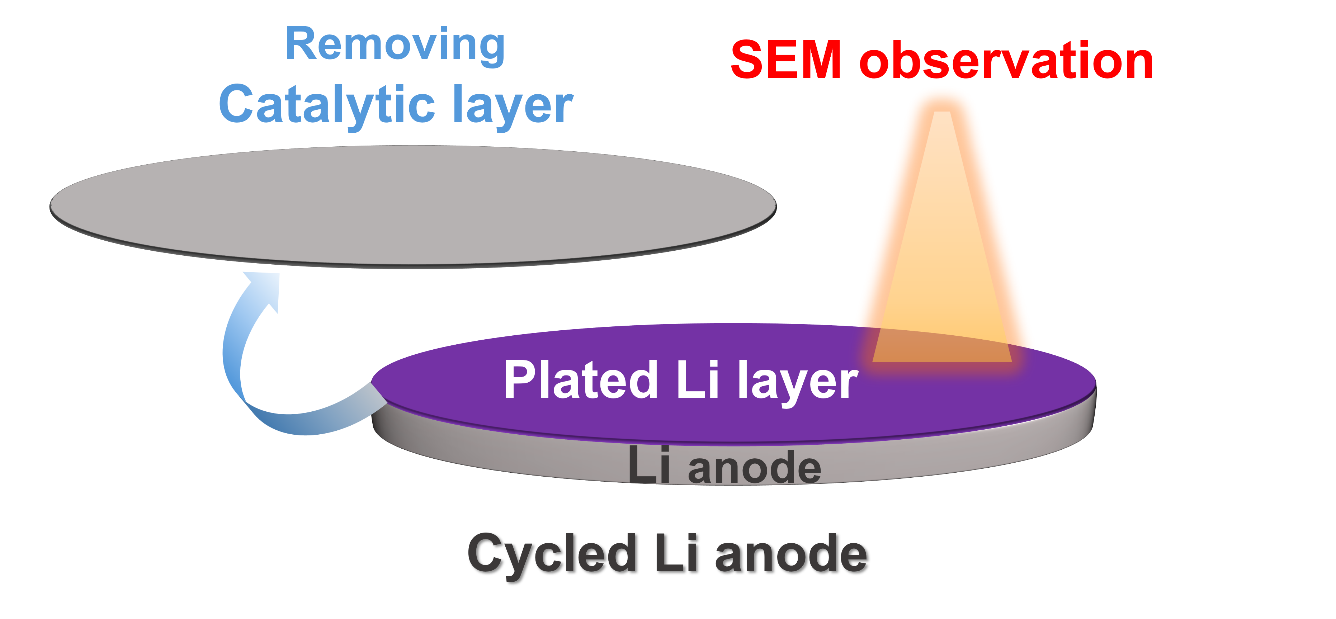


**Figure S55.** Schematics of the cycled SEM image of DEM-TMOs@C-Li electrode recorded from the top view.


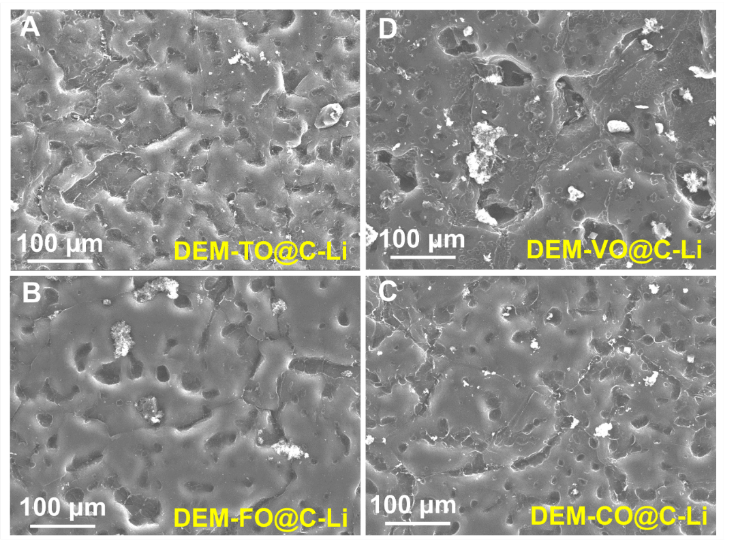


**Figure S56.** The SEM images of (A) DEM-TO@C-Li, (B) DEM-VO@C-Li, (C) DEM-FO@C-Li, (D) DEM-CO@C-Li from the symmetric cells at the end of the cycling for 600 h under -20°C.


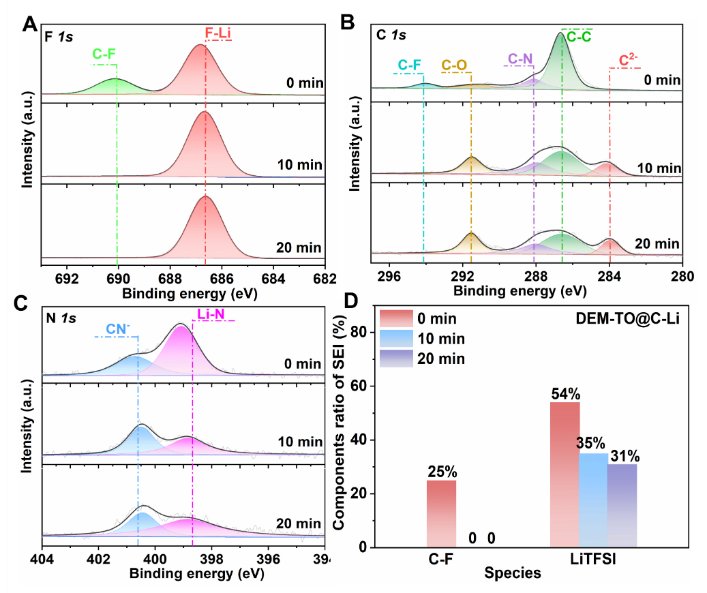


**Figure S57.** XPS depth profiles of SEI formed on the Li/electrolytes interface, including high-resolution (A) F *1s* and (B) C *1s* and N *1s* for DEM-TO@C-Li electrode at the end of cycling for 20 cycles at -20°C; (D) Comparison of component ratio of SEI layer on DEM-TO@C-Li electrode.


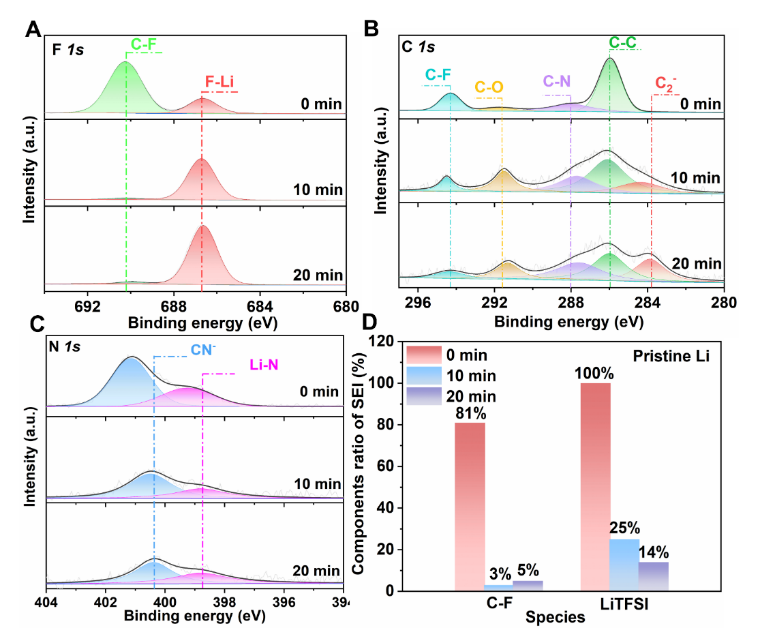


**Figure S58.** XPS depth profiles of SEI formed on the Li/electrolytes interface, including high-resolution (A) F *1s* and (B) C *1s* and N *1s* for pristine Li electrode at the end of the cycling for 20 cycles at -20°C; (D) Comparison of component ratio of SEI layer on pristine Li electrode.

.


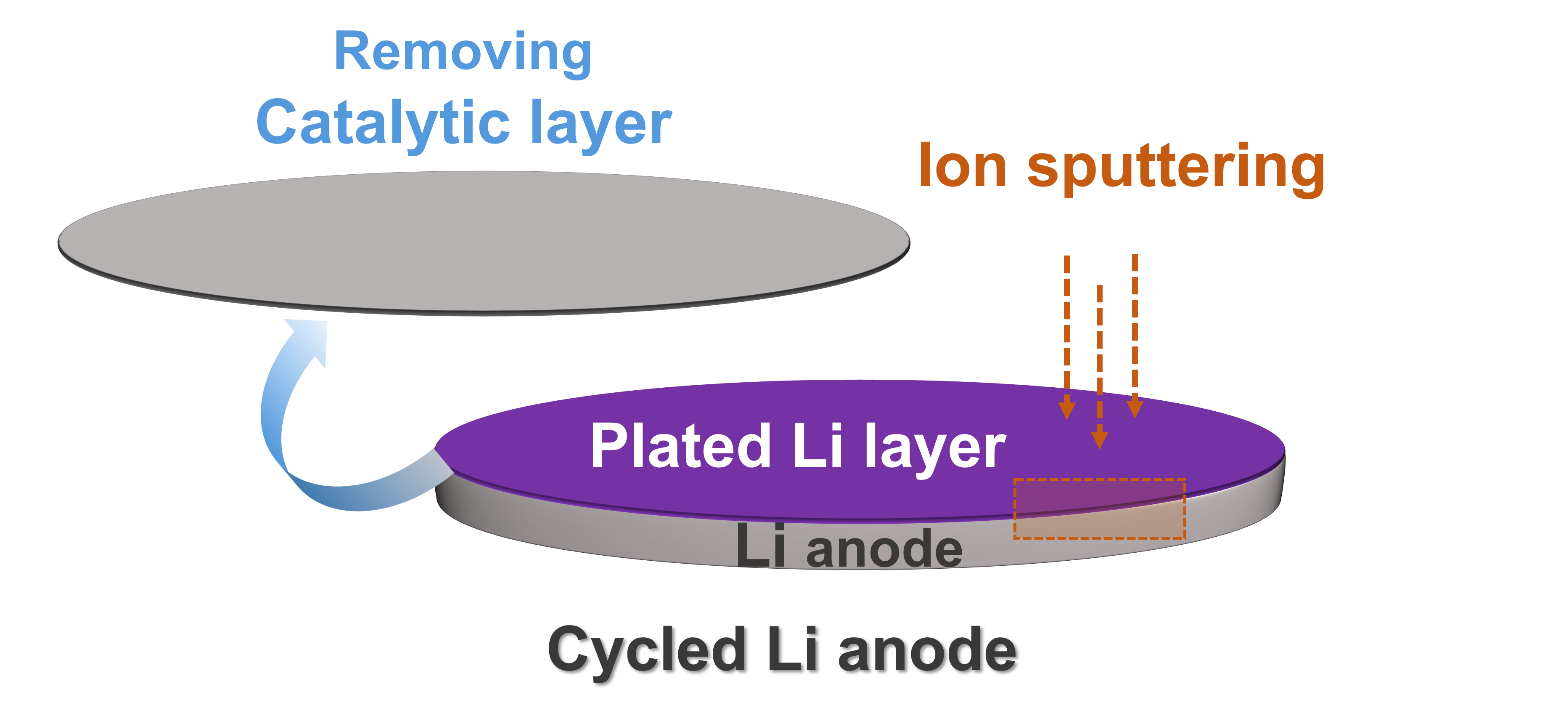


**Figure S59.** Schematics of the location for 3D morphology and species reconstruction of the DEM-TMOs@C-Li electrode via TOF-SIMS.


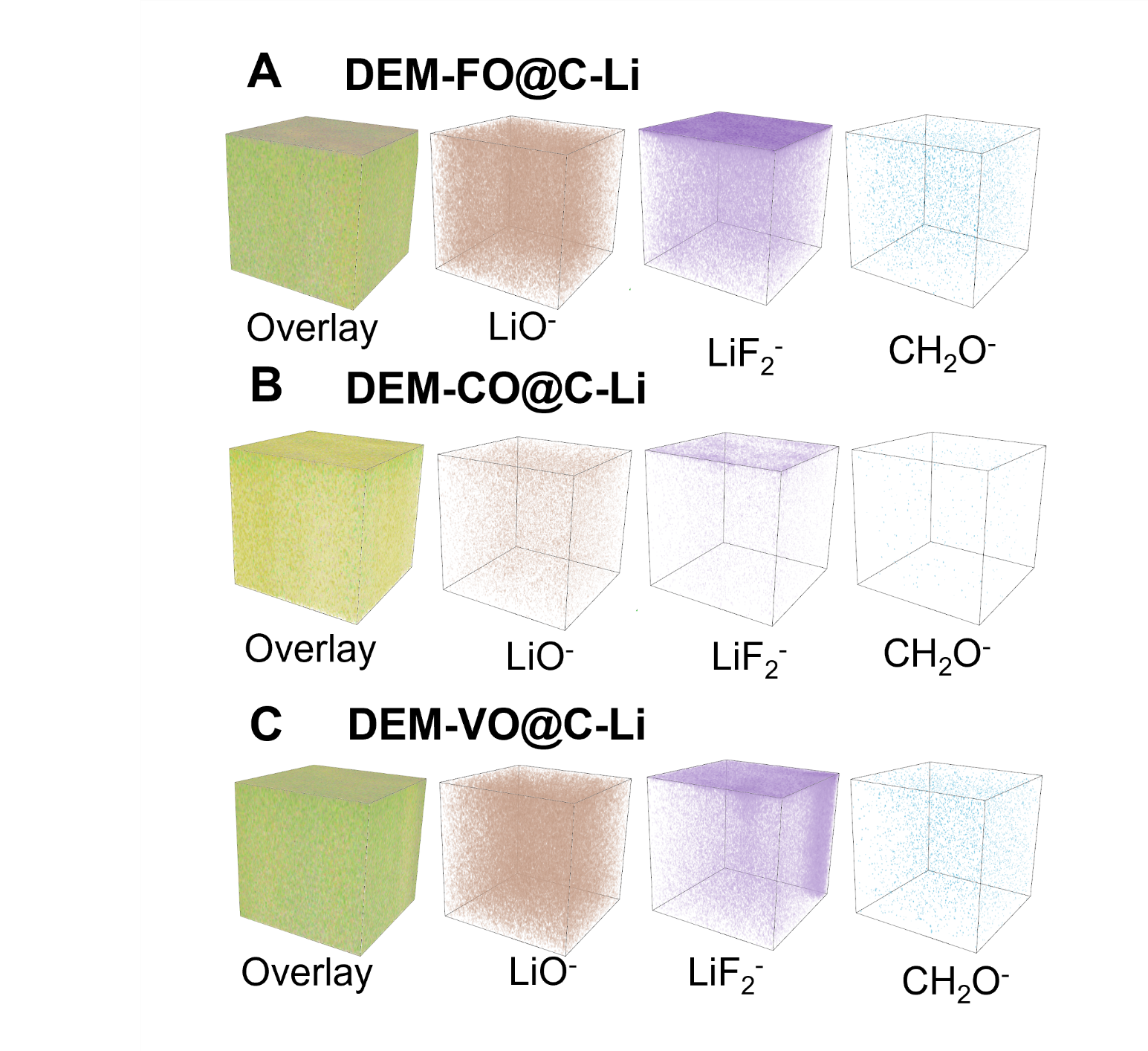


**Figure S60.** Interfacial 3D reconstruction of organic/inorganic species within interfacial SEI on (A) DEM-FO@C-Li, (B) DEM-CO@C-Li and (C) DEM-VO@C-Li, respectively.


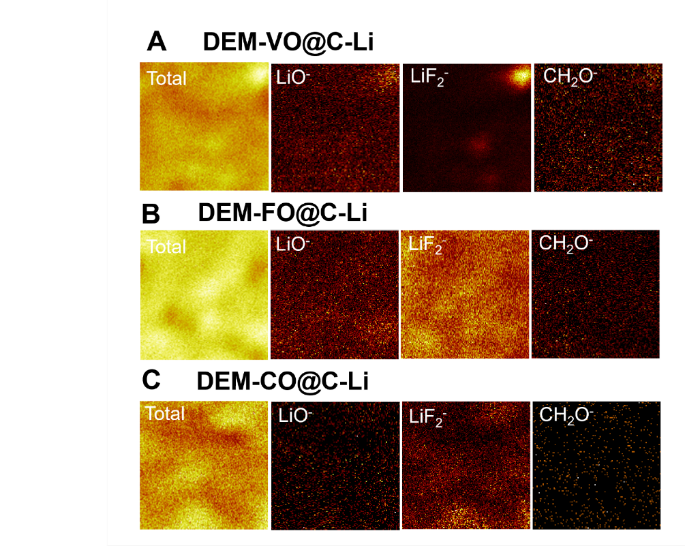


**Figure S61.** Surface 2D reconstruction of organic/inorganic species within interfacial SEI on (A) DEM-VO@C-Li, (B) DEM-FO@C-Li and (C) DEM-CO@C-Li, respectively.


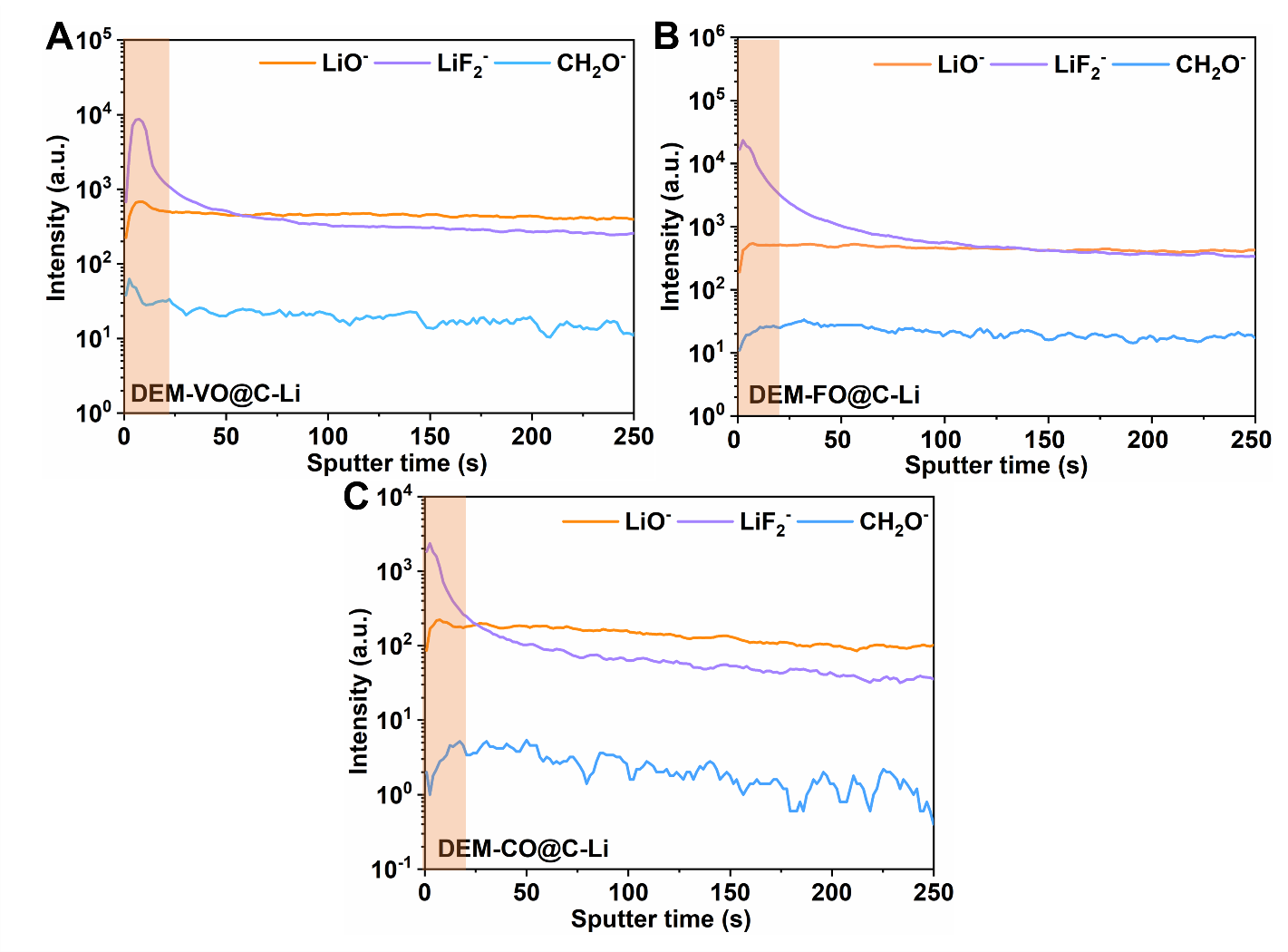


**Figure S62.** Intensity comparison of organic/inorganic species within interfacial SEI on cycled (A) DEM-VO@C, (B) DEM-FO@C and (C) DEM-CO@C modified Li, respectively.


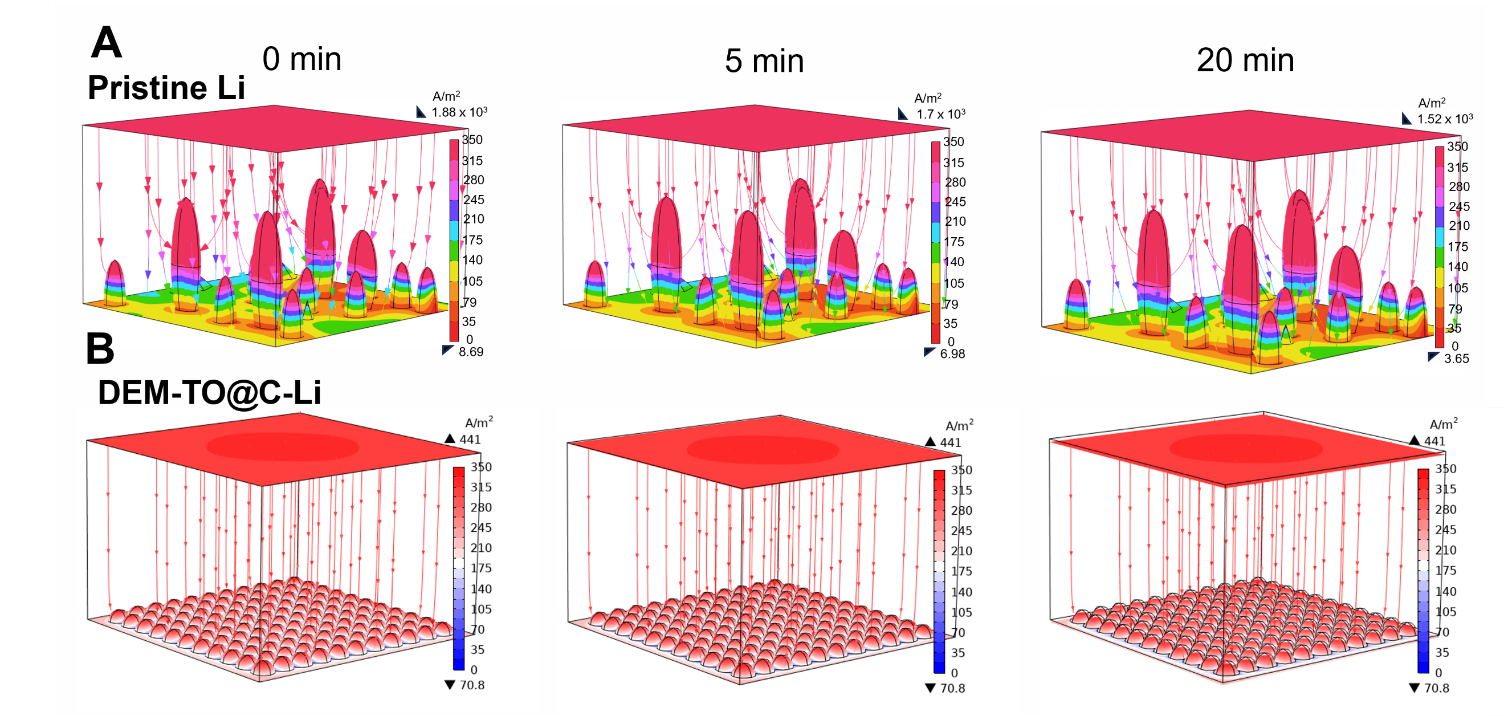


**Figure S63.** Current density distribution near the surface of (A) Pristine Li and (B) DEM-TO@C-Li anodes.

**Table S1.** Comparison of the ionic conductivity of various DEM-TMOs@C catalytic layer.

| Catalytic layer | Ion conductivity (mS cm^-1^) |
| --- | --- |
| DEM-TO@C | 2.94 |
| DEM-VO@C | 2.68 |
| DEM-FO@C | 2.42 |
| DEM-CO@C | 1.07 |

**Table S2.** The simulated parameters of EIS for the DEM-TMOs@C-Li measured under -20^o^C.

| Samples | Charge transfer resistance (Ω) | Slope (σ) |
| --- | --- | --- |
| DEM-TO@C-Li | 23 | 4.31 |
| DEM-VO@C-Li | 80 | 7.86 |
| DEM-FO@C-Li | 37.7 | 7.98 |
| DEM-CO@C-Li | 179 | 24.44 |
| Pristine Li | 2199 | 331 |

**Table S3** Comparison of electrochemical performance of the DEM-TO@C-Li anode with that in recent literatures.


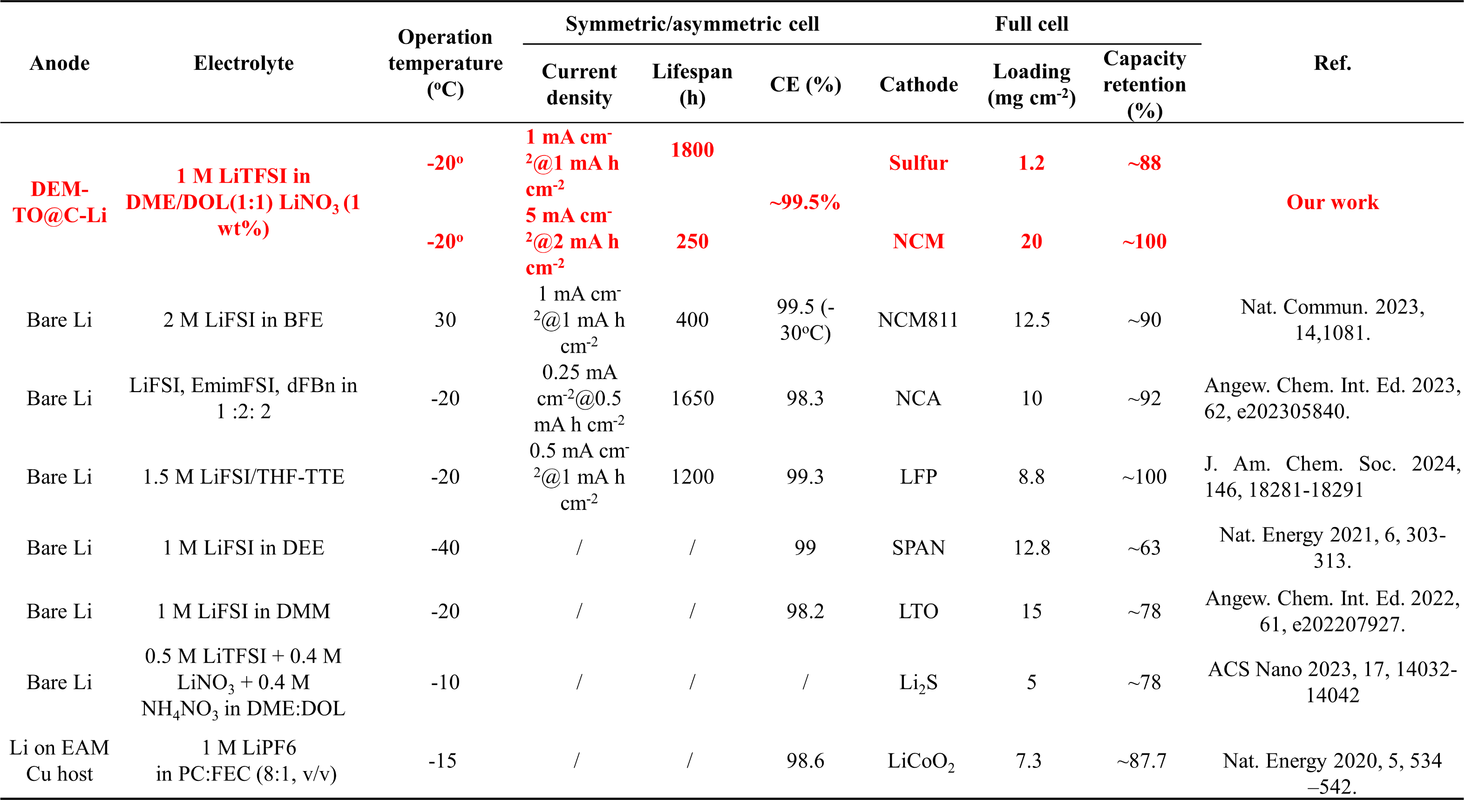


**References**

1. Kresse G, Hafner J. Ab initio molecular dynamics for liquid metals. *Phys. Rev. B* 1993, 47, 558-561.

2. Kresse G, Furthmüller J. Efficient iterative schemes for ab initio total-energy calculations using a plane-wave basis set. *Phys. Rev.* *B* 1996, 54, 11169-11186.

3. Smidstrup S, et al. QuantumATK: an integrated platform of electronic and atomic-scale modelling tools. *J. Phys-Condens. Mat.* 2019, 32, 015901.

4. Haas P, Tran F, Blaha P, Schwarz K. Construction of an optimal GGA functional for molecules and solids. *Phys. Rev. B* 2011, 83, 205117.

5. Li M, et al. Born–Oppenheimer molecular dynamics simulations on structures of high-density and low-density water: a comparison of the SCAN meta-GGA and PBE GGA functionals. *Phys. Chem. Chem. Phys.* 2021, 23, 2298-2304.

6. Shen Z, et al. Cation-doped ZnS catalysts for polysulfide conversion in lithium–sulfur batteries. *Nat. Catal.*‌‌ 2022, 5, 555-563.
